# Impacts of Lifestyle and Microbiota‐Targeted Interventions for Overweight and Obesity on the Human Gut Microbiome: A Systematic Review

**DOI:** 10.1111/obr.70037

**Published:** 2025-12-10

**Authors:** Yee Teng Lee, Ayça Akan, Dilara Beyza Önel, Evelyn Medawar, Daria E. A. Jensen, Arno Villringer, A. Veronica Witte

**Affiliations:** ^1^ Clinic for Cognitive Neurology University of Leipzig Medical Center Leipzig Germany; ^2^ Department of Neurology Max Planck Institute of Human Cognitive and Brain Sciences Leipzig Germany; ^3^ Berlin School of Mind and Brain Humboldt University of Berlin Berlin Germany

**Keywords:** gut microbiome, lifestyle interventions, obesity, randomized controlled trials

## Abstract

Obesity is intricately associated with the gut microbiome, and emerging research suggests that lifestyle interventions, such as dietary changes and active lifestyle, can significantly affect the composition and function of the gut microbiome. However, evidence demonstrating a causal link between these changes and long‐term weight loss or metabolic improvements remains limited. This systematic review investigates how overweight‐ and obesity‐targeted interventions, such as dietary modifications, physical activity, supplementation with prebiotics and probiotics, and fecal microbiota transplantation (FMT), manipulate gut microbiome diversity and composition, major metabolites, and weight status. We conducted a systematic literature search and included 87 out of 255 randomized clinical trials with 6086 adults aged 18–84 with a BMI ≥ 25 kg/m^2^. The quality of the included RCTs ranged from very low to moderate risk of bias. Most interventions did not cause any significant changes in microbial alpha or beta diversity, however, positive associations between prebiotic consumption and abundance of Actinobacteria and *Bifidobacterium* were observed, and intake of probiotics was related to increased levels of *Lactobacillus* and reduced body weight and body fat. We did not observe strong evidence for associations between SCFA levels, gut microbiome, and obesity. Overall, diversity and heterogeneity in reported outcomes, both in methods and results, were large. Taken together, our findings suggest that overweight‐ and obesity‐targeted dietary interventions of at least 4 weeks, particularly those involving prebiotics and probiotics, have the potential to beneficially alter the gut microbiome, although standardized protocols and harmonized reporting are needed to confirm this through meta‐analysis.

## Graphical abstract

Dietary interventions showed the strongest evidence for improving microbiome composition. Probiotics and prebiotics consistently increased beneficial bacteria. Exercise and fecal microbiota transplantation had limited evidence but showed potential for microbiome modulation.

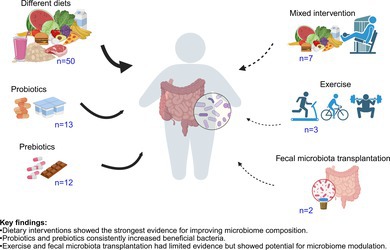

## 1 Introduction

Lifestyle interventions have been proposed as effective strategies for prevention and management of obesity. Of which, most interventions target different diet regimens and physical activity to improve obesity through weight loss. A recent study found that having a healthy diet and maintaining a regular exercise routine contributed to the reduction of the risk of getting cardiovascular diseases among adults with obesity [1]. As such, a recent systematic review reported that the combination of a customized hypocaloric diet complemented by strength and endurance exercises for at least 175 min per week was the most efficient in obesity management among adults with obesity [2]. The underlying mechanisms of how lifestyle interventions lead to improved obesity management are however not fully understood.

Recent research suggest that the development of obesity is influenced by the composition of microbial communities at various taxonomy levels in the gut, offering microbiome‐gut‐brain signaling as potential mediator or moderator of lifestyle effects on obesity [3, 4]. At phylum level, Bacteroidetes, Firmicutes, and Actinobacteria contribute to the pathophysiology of obesity [5]. It has been known that the Firmicutes/Bacteroidetes (F/B) ratio and the abundance of Firmicutes are higher among individuals living with obesity [6]. At genus level, a study reported that obesity was associated with a higher *Prevotella*/*Bacteroides* (*P*/*B*) ratio [7]. A recent study also reported a significant association between obesity and decreased microbial diversity and levels of certain microbial metabolites, such as short‐chain fatty acids (SCFAs) [8]. Kim et al. [9] showed that individuals with obesity and metabolic risk factors exhibited a lower *α*‐diversity than metabolically healthy individuals with obesity.

In parallel, emerging evidence has indicated that dietary patterns and physical activity regimens exert profound impacts on improving gut microbiome composition [10], which play a critical role in metabolic health [11]. Puljiz et al. [12] reviewed that dietary interventions, regardless of the duration, exert impacts on gut microbiome quickly. For instance, ketogenic diets increased *Akkermansia muciniphila* and reduced Firmicutes, which are associated with improved intestinal integrity, weight loss, and eventually better metabolic health, while Mediterranean diets raise the abundance of *Prevotella* and *Lachnospira* which are responsible for carbohydrate fermentation and subsequently SCFA production [13].

Excessive consumption of high‐calorie foods, particularly those high in added sugars and saturated fats, can increase the risk of obesity as the additional calories contribute to higher energy intake and fat accumulation in the body, which may negatively impact gut health by promoting the growth of harmful microbiome species [14]. On the contrary, intake of high‐fiber foods like fruits, vegetables, and legumes, which are the source of dietary fiber, has been associated with reduced weight gain and higher microbial diversity. A recent randomized clinical trial showed that high‐fiber and resistant starch diets increased daily calorie loss and reduced host metabolizable energy while increasing microbial 16S rRNA gene copy number and *β*‐diversity as well as fecal and serum SCFA levels compared to Western diets among young, healthy, and weight‐stable individuals [15].

Moreover, Noor et al. [16] reviewed that supplementation with probiotics, prebiotics, and synbiotics may manipulate the release of hormones and inflammatory factors that could influence gut microbiome and lead to weight changes. In brief, probiotics refer to food containing adequate amount of living microorganisms that are beneficial for health, such as yogurt. Prebiotics are nondigestible food ingredients like inulin that promote the growth of beneficial microorganisms in the gut. Synbiotics are the combinations of prebiotics and probiotics that synergistically improve the growth of beneficial microorganisms in the gut. A meta‐analysis of 11 studies also showed that consumption of probiotics could improve obesity measures and modulate gut microbiota in patients with obesity undergoing bariatric surgery [17 ].

Moreover, probiotic strains like *Bifidobacterium* and *Lactobacillus* increase the production of SCFAs, which contribute to better lipid metabolism and further downstream effects like reducing hyperlipidemia [18]. SCFAs have been suggested to protect against weight gain by strengthening appetite control and raising energy expenditure through activating free‐fatty acid (FFA) receptors in the hypothalamus [19]. Furthermore, SCFAs are important gut metabolites that help strengthen gut barrier function and produce intestinal epithelial cells. SCFAs may also act as signaling molecules and play a role in intestinal G‐protein‐coupled receptor activation, which subsequently involve in the secretion of gut hormones that are important in the treatment of obesity [20].

Despite the homeostatic mechanisms, recent research also suggests that dietary fiber may modulate brain reward circuitry. A randomized controlled trial using inulin supplementation found reduced reward‐related brain activation patterns related to food motivation, which were correlated with increased Actinobacteria abundance and enhanced SCFA‐producing pathways [21]. This highlights an additional pathway through which fiber intake may contribute to energy balance and weight regulation via reward‐related processing.

In sum, current research indicates that diet‐related and exercise interventions manipulate the gut microbiome at various levels and play a role in obesity development and weight management. However, due to diverse study designs and the different levels of reporting, knowledge on the exact microbial changes that reliably occur after different lifestyle interventions in obesity remains obscure. The primary objective of this pre‐registered systematic review is thus to determine causal effects of lifestyle and microbiota‐targeted interventions on the gut microbiome among adult populations with overweight or obesity based on available randomized controlled trials. For the primary outcome measure, we considered changes in gut microbiome, including microbial diversity, microbiome composition, and microbial metabolites after the intervention compared to placebo. For the secondary outcome, we assessed the correlations between gut microbiome and obesity‐related outcomes.

## 2 Methods

### 2.1 Research Strategy and Registration

We utilized PICOS strategy to perform a thorough literature search. The PICOS strategy: Population (P), Intervention (I), Comparison (C), Outcome (O), Study design (S) was assumed to determine the eligibility criteria (Table 1). The literature search was carried out through the scientific database PubMed in September 2021, which resulted in 210 hits. The time scope of the search was 10 years (2011–2021), and the language of the literature was strictly English. To incorporate the latest evidence, an extended search using the same search strategy was performed through PubMed in July 2024, capturing studies published up to 31st May 2024. The extended literature search resulted in 45 hits.

**TABLE 1:** PICOS framework showing the keyword selection process and search strategy.

Inclusion criteria	Descriptions
Population	Overweight, obesity, adiposity
Intervention	Diet therapy, fecal microbiota transplantation, exercise, probiotics, prebiotics, weight loss
Comparison	Diet maintenance, different diet types, placebo, different biotics, sedentary control, different types of exercise
Outcome	Gut microbiome, gut microbiota
Study design	Clinical trial, randomized controlled trial

For the search strategy, MeSH (Medline) and free terms were combined via using the Boolean operators “OR” and “AND” (File S1). MeSH and similar free terms were cross‐evaluated, and the term that covered all plus more outputs was assumed. These terms used in the search were: “Gut microbiome”, “Gut microbiota”, “Overweight”, “Obesity”, “Adiposity”, “Diet therapy”, “Fecal microbiota transplantation”, “Exercise”, “Probiotics”, “Prebiotics”, “Weight Loss”, “Intervention”. The full PubMed search query and all applied filters (i.e., language, age, study design) can be found in Supplementary File 1. The present systematic review followed the PRISMA checklist [22] (Table S1) and was pre‐registered in the International Prospective Register of Systematic Reviews (PROSPERO) under the number: CRD42021281444 (https://www.crd.york.ac.uk/prospero/display_record.php?ID=CRD42021281444).

While our preregistered protocol stated that we would search PubMed, MEDLINE, and the Cochrane Library, the final search was conducted using only PubMed. A preliminary search showed that PubMed returned the same set of relevant studies identified in MEDLINE based on our specific inclusion criteria, and the Cochrane Library was excluded due to access limitations. Therefore, additional database searches were deemed unlikely to identify further eligible studies.

### 2.2 Eligibility Criteria

Randomized controlled trials (RCTs) of lifestyle and microbiota‐targeted interventions that evaluated the gut microbiome composition of adult humans with body mass index (BMI) over 25 kg/m^2^ were included. This BMI criterion enabled us to include a wide range of studies investigating obesity, overweight, or both. Studies including animal models, infants, children, teenagers; populations with serious mental or physical diseases (i.e., diabetes, cancer); invasive or nonlifestyle interventions (i.e., surgery, antibiotic treatment), and articles without a gut microbiome outcome were excluded. Studies conducting fecal microbiota transplantation (FMT) were also included as FMT aimed to boost the growth of beneficial microbiota.

### 2.3 Assessment of Study Quality and Risk of Bias

To assure the study quality, Grading of Recommendations, Assessment, Development and Evaluation (GRADE) approach was utilized [23]. This approach consisted of evaluations of five different domains within a randomized controlled trial to determine the risk of bias in each of them: Randomization, allocation concealment, blinding, loss to follow up, and other causes of bias. Each study was scored across five bias domains using a numeric system: + = 1 (low risk), ? = 0.5 (unclear), and – = 0 (high risk). Blinding (S = single‐blind, D = double‐blind) was noted but not scored. Total scores (max 5) were categorized as: ≤ 1 (very low quality), 1.5–2.0 (low), 2.5–3.0 (moderate), and ≥ 3.5 (high quality). The article could receive a plus for each domain if the adequate criteria was met, obtaining a maximum of four pluses in total for high‐quality. Articles that had low or very low quality according to the GRADE approach were excluded due to high risk of bias. In line with Dettori [24], if the dropout rate was less than 5% it was considered as not a threat to validity, whereas > 20% was considered as a serious threat when evaluating for attrition bias. As per the GRADE guidelines, we also evaluated the reasons to drop out for studies that had rates higher than 5% to come to a definitive scoring for the attrition bias. Although our preregistered protocol indicated the use of the Revised Cochrane risk‐of‐bias tool (RoB 2.0), we ultimately employed the GRADE approach as it provided a more comprehensive and flexible framework to evaluate both individual study risk and overall strength of evidence, which aligned better with the aims of this review. While our protocol allowed for exclusion of studies with low or very low quality, no studies were excluded based on this criterion as all included studies met at least a moderate level of quality.

### 2.4 Data Extraction and Synthesis

**FIGURE 1:**
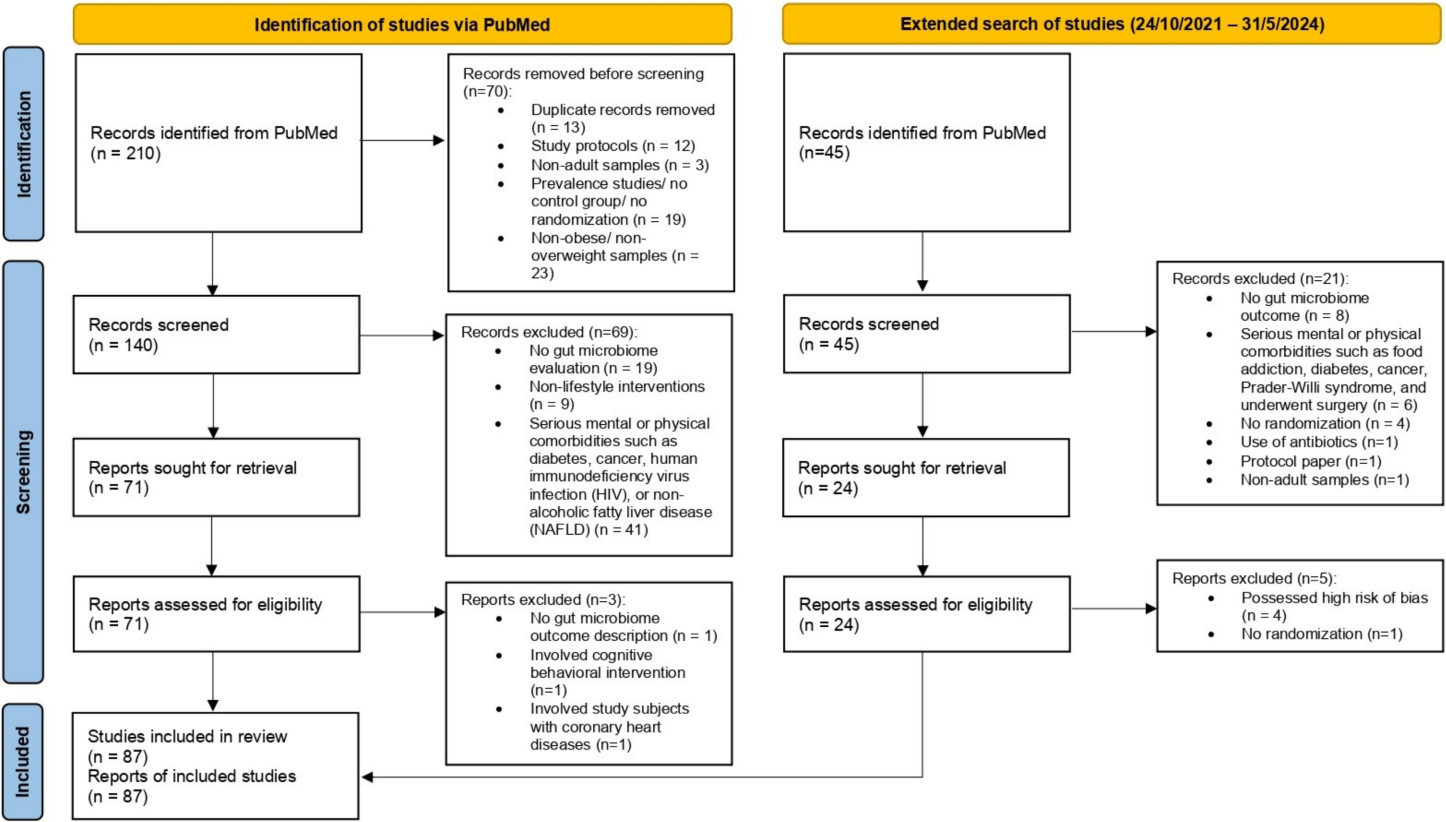
PRISMA flow diagram showed the study selection process of lifestyle and microbiota‐targeted interventions for obesity.

A two‐stage selection process was assumed for data extraction: (1) screening titles and abstracts, (2) full‐text screening. A PRISMA (Preferred Reporting Items for Systematic Reviews and Meta‐analyses) flow‐chart was utilized (Figure 1). For the full‐text screening, three review authors (A.A., D.O., and Y.T.L.) evaluated each study to assess whether predefined selection criteria were met. Extracted data from the included studies were summarized in tables, with the following information: study identification (author, year), study description, sample size, sample description, types of intervention, outcomes related to obesity measures, and gut microbiome‐related outcomes (see results). For obesity‐related outcomes, we assessed changes in body weight, body fat, and anthropometric measurements such as waist and hip circumferences and waist‐to‐hip ratio. Regarding microbiome‐related outcomes, we evaluated the changes in taxa abundance, *α*‐ and *β*‐diversity analyses, and levels of microbial metabolites such as SCFAs. Specifically, we reported only statistically significant results in our studies. Due to the heterogeneity and incomparability of interventional categories and reported microbial outcomes, a meta‐analysis was not conducted.

## 3 Results

### 3.1 Study Selection and Characteristics

A total of 210 articles were obtained from the initial database searching from September 2011 to September 2021, while an additional 45 articles were later retrieved from the extended search from October 2021 to May 2024. After the preliminary screening, 183 full‐text articles were carefully evaluated. Finally, 87 relevant articles that fulfilled the pre‐defined inclusion and exclusion criteria were included in the final systematic review (Figure 1). Studies that did not assess gut microbiome outcomes, carried out non‐lifestyle‐related interventions, or involved study subjects with serious mental and physical comorbidities had been excluded.

Overall, data from 6086 adults whose ages ranged from 18 to 84 with a BMI of 25 kg/m^2^ or higher were analyzed. Of 87 studies, 53% (*n* = 46) examined combined overweight/obesity, 23% (*n* = 20) focused on obesity alone, 22% (*n* = 19) on overweight alone, and 2% (*n* = 2) compared lean versus overweight populations. The sample size of the included studies ranged from 10 to 400, and the intervention periods of the studies ranged from 2 weeks to 1.5 years. The vast majority of studies reported on diverse dietary interventions (*n* = 50), such as grain diet and supplementation (*n* = 8), protein supplementation (*n* = 3), dairy product consumption (*n* = 2), mixed diets (*n* = 18) and additional food supplementation (*n* = 19), followed by probiotics (*n* = 13), prebiotics (*n* = 12), mixed interventions (*n* = 7), interventions with exercise (*n* = 3), and FMT (*n* = 2) (Table S2). Notably, not all intervention studies were intended to induce weight loss.

Among the 50 studies that reported the effects of dietary interventions (Table S2a–c), most of the grain diet studies (*n* = 8) compared the whole grain products against refined grain. The studies that used dairy product supplements (*n* = 2) compared different kinds and concentrations of milk, while protein‐supplemented studies compared plant protein, animal protein, and maltodextrin as well as protein products with different concentrations. A total of 18 studies compared different dietary patterns, such as Mediterranean diets, New Nordic diet, high‐protein diets, fiber‐enriched diets, calorie‐restricted diet, high‐dairy diet, low‐carbohydrate diet, different‐meat diets, fish intake and vegan diet to contrasting dietary regimens. There was also a study that involved five intervention groups to compare high‐fat diets with high carbohydrate diets (Table S2b; [25]). In addition, there were several studies (*n* = 19) that studied the effects of supplementing different dietary contents to the normal diet, including the intake of high calcium, raw almond snacks, fresh kimchi, consumption of vitamin D, etc.

Apart from those studies, 13 probiotic studies reported on the intervention effect of probiotic strains compared to placebo supplements, which were mainly maltodextrin and cellulose without probiotic strains. Twelve prebiotic studies mainly used oligosaccharides and inulin in comparison with maltodextrin, cellulose, or polyunsaturated fatty acids as controls. Several studies compared the effects of various kinds of interventions (*n* = 7). For instance, Rajkumar et al. [26] compared the effects of probiotics against omega‐3 supplements, while Gutiérrez‐Repiso et al. [27] investigated the effects of synbiotics with either a very‐low‐calorie ketogenic diet or a low‐calorie diet. Also, there were three studies that reported exercise intervention with different types and intensities: one implemented aerobic exercise via habitual cycling [28], one involved supervised resistance (strength) training [29], and another combined high‐intensity interval training with resistance training [30]. Additionally, two studies compared the effects of FMT intervention in people with obesity using different comparators. Yu et al. [31] used an active placebo (cocoa/gelatin mixture), while Zhang et al. [32] employed placebo FMT capsules (microcrystalline cellulose). Both studies sourced FMT from healthy lean donors.

### 3.2 Sequencing Methods and Output

Among the 87 studies, 71 utilized 16S rRNA or rDNA gene sequencing (hereafter 16S); some studies (11%, *n* = 10/87) analyzed the microbial profiles with a combination of two sequencing methods. The most used primer for 16S sequencing was V3‐V4, which amplified V3‐V4 hypervariable regions. Besides, six studies used shotgun metagenomic sequencing, 2 utilized real‐time PCR, 1 used microarray, 1 utilized fluorescence in situ hybridization (FISH) analysis, and the remaining 6 did not report the sequencing method used.

Overall, a total of 48% studies binned sequencing data into operational taxonomic units (OTUs; *n* = 42/87 studies), followed by amplicon sequence variants (ASVs) (16%; *n* = 14/87 studies), and molecular operational taxonomic units (MOTUs) (1%; *n* = 1/87 studies). A total of 30 studies (34%) did not report the type of taxonomic units utilized in the studies. Besides, most studies reported the microbial results at genus (44%, *n* = 38/87 studies) and species (47%, *n* = 41/87 studies) levels. Going up the taxonomy, 6 studies reported microbial results at phylum level and 3 studies reported at family level.

### 3.3 Study Findings

#### 3.3.1 Primary Outcome Measures: Changes in Microbial Diversity

**FIGURE 2:**
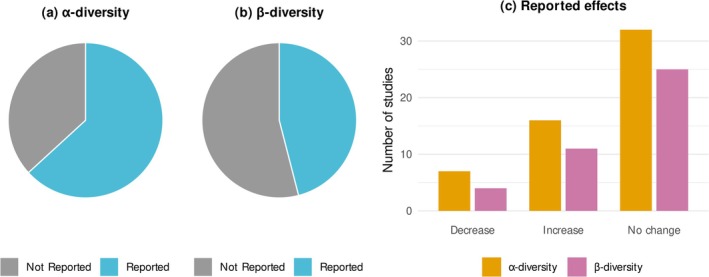
Number of studies that conducted (a) *α*‐ and (b) *β*‐diversity analyses and (c) reported effects of studies that reported changes in both *α*‐ and *β*‐diversity indices.

**FIGURE 3:**
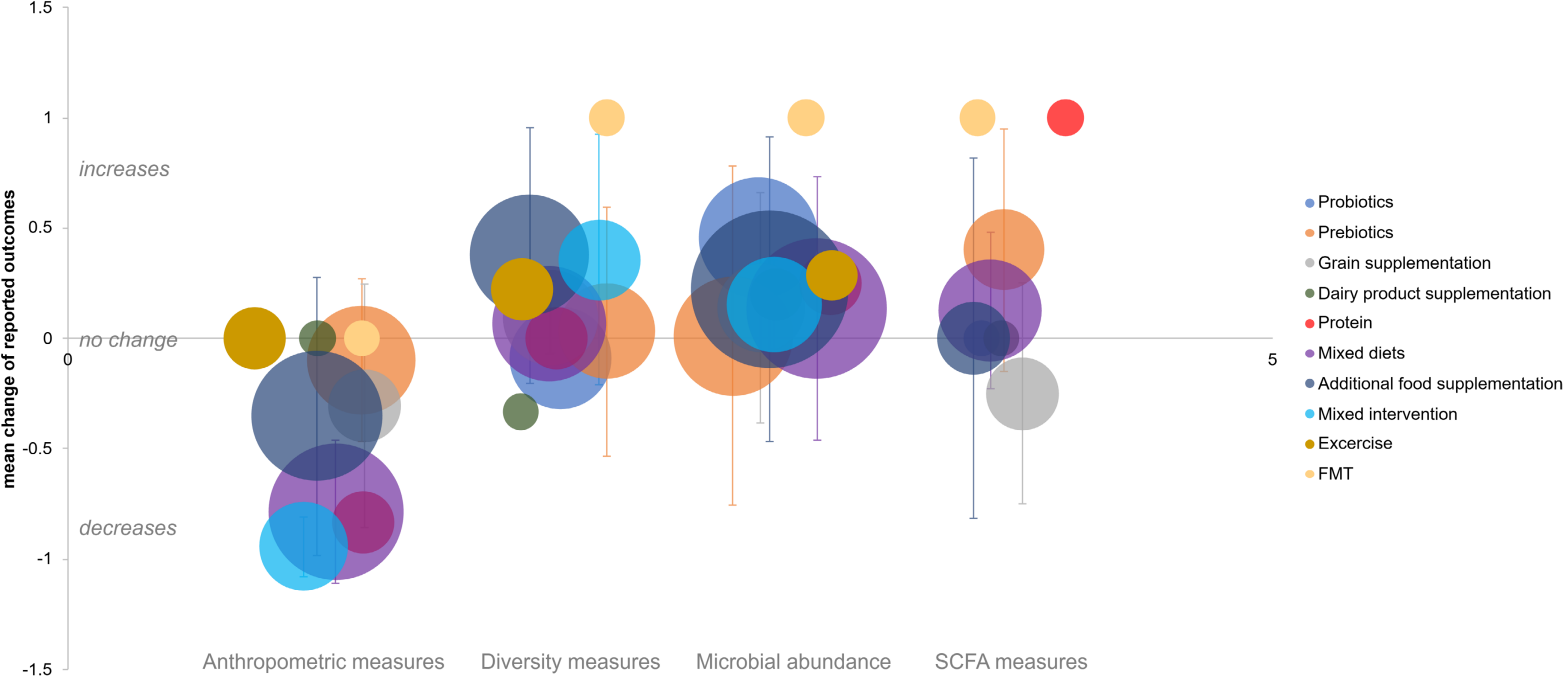
The bubble chart illustrates how different interventions impact human gut microbiome outcomes. The overall reported effects of different intervention categories across anthropometric measures (body weight, body fat etc.), diversity measures, microbial abundance, and short‐chain fatty acids (SCFAs) measures were computed based on the mean reported changes across studies. Each bubble represents an intervention category, with the bubble size corresponding to the number of studies. The standard deviations, as shown by the vertical error bars, indicate the variability of each intervention group for each outcome measure.

As shown in Figure 2a,b, 63% of the included studies reported *α*‐diversity analyses and 46% reported *β*‐diversity analyses, and 34% of studies did not report any diversity measure. A total of 83 *α*‐diversity analyses were conducted across 55 studies (*n* = 55/87 total studies) (Figure 3; Table S3). Reported *α*‐diversity metrics were diverse, including measures of richness, Shannon index, abundance‐based coverage estimator (ACE), Chao1, Simpson index, etc. The most reported *α*‐diversity metric was Shannon index (33% of total analyses; *n* = 38/115 analyses). Most of the analyses reported no difference in *α*‐diversity (58%; *n* = 32/55 reported studies), while 29% studies reported increased *α*‐diversity (*n* = 16/55 reported studies) and 13% studies demonstrated reduced *α*‐diversity (*n* = 7/55 reported studies).

Besides, 46 *β*‐diversity analyses were reported across 40 studies (*n* = 40/87 total studies). The reported *β*‐diversity metrics were Bray–Curtis index, weighted and unweighted UniFrac distance metrics, Aitchison distance metric, and Morisita‐Horn distance matrix. Of which, most studies used UniFrac distance (60% of total analyses; *n* = 29/48 analyses). A total of 25 studies showed no change in *β*‐diversity, 11 showed increased *β*‐diversity, and 4 showed reduced *β*‐diversity.

Despite that most studies reported null results for diversity measures across the categories (Figure 2c), two probiotic intervention studies reported increases in *α*‐diversity [33, 34] while Sergeev et al. [35] showed reduced *α*‐diversity. Four prebiotic studies showed reduced *α*‐diversity indices [21, 36, 37, 38]. Only one probiotic study showed reduced *β*‐diversity [39], while one prebiotics study reported increased *β*‐diversity [21].

Across dietary interventions, grain intake (*n* = 2) showed increased *α*‐diversity ([40] [16S rRNA]; [41] [16S rRNA]) while a dairy intervention (i.e., soymilk) reported a reduction in *α*‐diversity (*n* = 1) ([42] [16S rRNA]). None of the studies involving protein interventions showed changes in both *α*‐ and *β*‐diversities ([43] [16S rDNA]; [44] [16S rRNA and shotgun metagenomics], [45] [16S rRNA]). Among the mixed diet interventions, both calorie‐restricted high protein diet ([46] [16S rRNA]) and Mediterranean diet ([47] [16S rRNA]) increased *α*‐diversity compared to the calorie‐restricted normal protein diet and the Western diet respectively. Intriguingly, vegan diet did not change *α*‐diversity in Kahleova et al. [48] [16S rRNA] while the microbial richness was increased in both fried and boiled meat groups ([49] [16S rRNA]).

Two exercise interventional studies showed an increase in *α*‐diversity ([29] [16S rRNA]; [28] [16S rRNA]) while the remaining study showed unchanged *α*‐diversity but increased *β*‐diversity ([30] [16S rRNA]). Surprisingly, Yu et al. [31] [16S rRNA] demonstrated that FMT did not affect the microbiome diversity despite that the microbiome composition of the FMT recipients was shifted toward the microbiome of the FMT donors. On a contrary, Zhang et al. [32] [16S rRNA] showed increases in both *α*‐ and *β*‐diversity indices.

Overall, the systematic review yielded inconclusive results regarding the diversity measures due to a scarcity of reported data across most studies. It was noted that many studies employed non‐homogeneous diversity measures, and the outcomes suggested that there was no intervention effect on the changes in microbial diversity.

#### 3.3.2 Changes in Abundance of Microbiota

Relative or absolute microbial abundance at various taxonomic ranks were reported in most studies. At phylum level, the most reported phyla across all studies were Actinobacteria, Bacteroidetes, Firmicutes, and Proteobacteria. Of all the categories, it was shown that prebiotic interventions increased Actinobacteria (*n* = 5/6 reported studies). Interestingly, the consumption of Korean food elements such as kimchi (Korean fermented cabbage), Schisandra chinensis fruit, and Bofutsushosan herbal extract caused increases in the abundance of Bacteroidetes and reduction in Firmicutes [50, 51, 52].

At family level, microbial changes of *Lachnospiraceae* (*n* = 18 reported studies) and *Ruminococcaceae* (*n* = 18 reported studies) were inconsistent across the interventions. At genus level, genera *Bacteroides* (*n* = 25 reported studies) and *Bifidobacterium* (*n* = 24 reported studies) were most reported. Across the interventions, both prebiotic (*n* = 7/8 reported studies) and probiotic interventions (*n* = 4/4 studies) consistently demonstrated increased abundance of *Bifidobacterium*. Moreover, probiotic interventions also significantly increased the abundance of *Lactobacillus* (*n* = 5/6 reported studies) along with *Bifidobacterium*.

Few studies also reported the changes in Firmicutes/Bacteroidetes (F/B) (*n* = 11 reported studies) as well as *P*/*B* ratios (*n* = 3 reported studies) (Table 2; Table 3). Of which, F/B ratio either remained unchanged (45%; *n* = 5/11 reported studies) or reduced (55%; *n* = 6/11 reported studies) after the interventions. Out of the five studies that measured *P*/*B* ratios, only two studies showed the results of *P*/*B* ratios after intervention, where one showed increased *P*/*B* ratio [59] while the two studies showed stable *P*/*B* ratio [58, 60]. Taken together, the limited number of studies available on the changes of both F/B and *P*/*B* ratios uncovered varied outcomes.

**TABLE 2:** Studies that reported Firmicutes/Bacteroidetes (F/B) ratio (*n* = 11).

Intervention	Studies	F/B ratio
Probiotics	[53]	Decrease
Prebiotics	[45]	No change
Grain	[41]	No change
Mixed diet	[48]	No change
	[54]	Decrease
	[55]	Decrease
Additional food supplementation	[56]	Decrease
	[57]	Decrease
Mixed intervention	[27]	No change
	[58]	No change
	[52]	Decrease

**TABLE 3:** Studies that reported *Prevotella*/*Bacteroides* (*P*/*B*) ratio (*n* = 3).

Intervention	Studies	*P*/*B* ratio
Mixed diet	[59]	Increase
	[60]	No change
Mixed intervention	[58]	No change

#### 3.3.3 Changes in Levels of Short‐Chain Fatty Acids (SCFAs)

**FIGURE 4:**
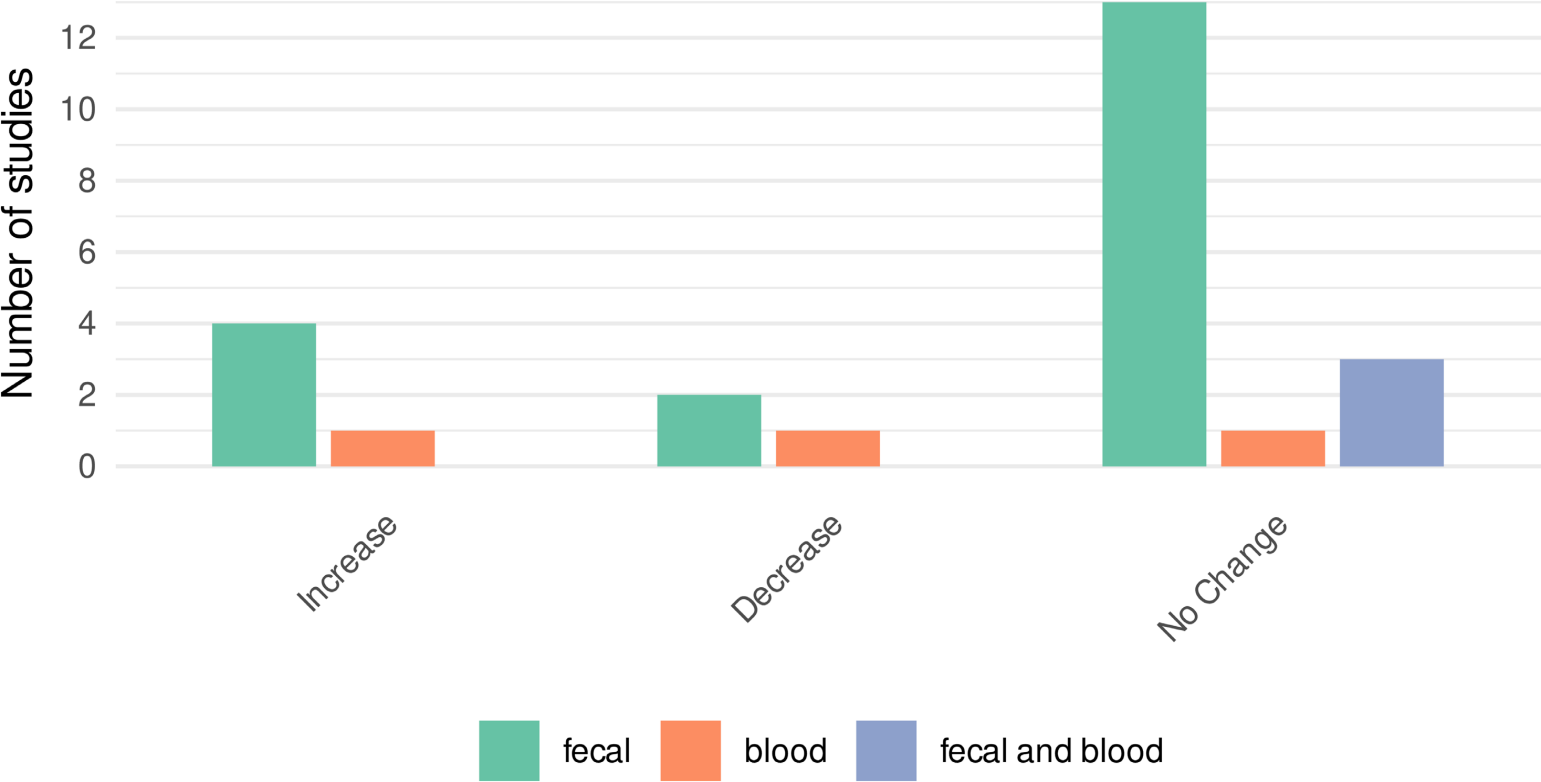
Number of studies that reported the changes in SCFA levels from various sources.

A total of 25 studies (29%) reported the change in SCFA levels after intervention, of which most of them reported fecal SCFA levels (*n* = 19), 3 reported SCFA levels in blood samples, and 3 measured both fecal and plasma SCFA levels (Figure 4). The results of SCFA change were heterogeneous across the interventions and within subcategories. For instance, 2 out of 5 prebiotic studies reported increased SCFA levels [38, 61], while the others showed no change [21, 36, 62]. Within the grain intervention, only one study demonstrated reduced SCFA levels [63], while the remaining three reported studies showed unchanged SCFA levels [40, 41, 64].

#### 3.3.4 Changes in Body Weight, Body Fat, and Anthropometric Measures

A total of 27 studies reported reduction in body weight, while 29 studies reported no change after respective interventions. Of which, 44% of the probiotic studies showed significant reductions in body weight after the interventions, while 56% of the probiotic studies reported no change (*n* = 5/9 reported studies). 88% of the prebiotic studies (*n* = 7/8 reported studies) reported no change in body weight, while 71% of the mixed dietary interventions (*n* = 10/14 reported studies) showed reductions in body weight. Besides, 23 studies reported reduced body fat, while one study demonstrated an increase in body fat after the prebiotic intervention [21]. Meanwhile, 20 studies demonstrated reductions in various anthropometric measures including waist circumference, waist‐to‐hip ratio, etc., while 17 studies reported no change after respective interventions.

With respect to the consumption of protein, one of the protein‐supplemented studies showed a significant reduction in visceral fat [44], and another study demonstrated that the high protein diet induced a significant reduction in body weight and waist and hip circumferences after the intervention [65]. Also, 4 mixed diet studies demonstrated significantly reduced body weight in low carbohydrate [65, 66] and plant‐based diets [48, 67], despite that the intervention periods varied across the studies. Meanwhile, Marungruang et al. [59] showed that multifunctional diets including plant‐based food component and fish also reduced body weight compared to pure plant‐based diet.

Interestingly, one interventional study with exercise showed a significant decrease in fat mass in all intervention groups [28], while two other exercise studies demonstrated increased body weight after supervised training programs for 12 [30] and 6 weeks [29] respectively. However, Dupuit et al. [30] showed a reduction in anthropometric measures while Cullen et al. [29] showed an increase in anthropometric measures. Among the FMT studies, only Yu et al. [31] reported anthropometric outcomes which showed no significant changes in body weight or body fat following the intervention.

#### 3.3.5 Association Between Obesity and Microbiome Measures After Interventions

Hjorth et al. [68] showed that the baseline *P*/*B* ratio was positively associated with weight loss after a Nordic dietary intervention. This was similarly seen in two other studies [69, 70]. Besides, several studies in this systematic review reported the change in abundance of certain microbiota was associated with the change in waist circumference. For instance, Lee et al. [52] showed the association between increased abundance of Gram‐negative bacteria and raised waist circumference, while the higher abundance of *Prevotella* [70], *Akkermansia* [71], and *Bifidobacterium* [50] were associated with reduced waist circumference. A study also showed that a higher abundance of Actinobacteria was related to a lower body fat [50].

### 3.4 Quality Assessment

Risk of bias was considered *very low* in 47 studies, *low* in 21 studies, and *moderate* in 19 studies (Table S4). Of which, 6 studies on probiotics and 7 studies on prebiotics scored *very low*. 58% of the dietary interventions scored *very low* (*n* = 29/50), with studies in the additional food supplement subcategory demonstrating *very low* risk of bias (*n* = 11), followed by mixed diets (*n* = 9), grain (*n* = 6), protein (*n* = 2), and 1 from dairy product intervention. Besides, 3 out of 7 mixed interventions scored *very low* risk of bias, while all FMT studies were rated as *very low* risk of bias (*n* = 2). Lastly, each of the exercise studies scored *low* and *moderate* risk of bias respectively (*n* = 3). The major sources of potential bias were selection bias due to unclear randomization, inadequate information about allocation concealment, and attrition bias due to incomplete outcome disclosure in the publications. Notably, the blinding procedure in the included studies was also primarily documented and reported by the respective authors.

Most studies with *moderate* risk of bias provided insufficient information about randomization of participants and unclear allocation concealment. Blinding of participants and researchers were also unclear. Other than that, studies with a *very low* risk of bias had a higher number of study participants, ranging from 24 to 400. In contrast, studies with *low* and *moderate* risks of bias had between 20 and 134 and between 17 and 135 participants, respectively. Additionally, it was observed that the length of the interventional periods varied across studies with different risks of bias. For studies with a *very low* risk of bias, the periods ranged from 2 weeks to 14 months. For those with a *low* risk of bias, the periods ranged from 4 weeks to 50 weeks, and for studies with a *moderate* risk of bias, the periods ranged from 2 weeks to 1 year. Noticeably, most of the studies preregistered their RCTs (*n* = 79), while the remaining eight studies without preregistration had higher risk of bias.

## 4 Discussion

In this systematic review, we included 87 studies reporting the association between microbiome and overweight/obesity through lifestyle and microbiota targeted interventions based on RCT design. The study analyzed data from 6086 adults with a BMI of 25 kg/m^2^ or higher and examined interventions with prebiotics, probiotics, diverse dietary interventions such as protein supplementation, grain supplementation, and dairy product consumption, mixed diets, mixed interventions, exercise interventions and FMT, ranging in duration from 2 weeks to 1.5 years. The assessment of risk of bias revealed that most of the dietary intervention studies possessed *very low* or *low* risk. Overall, the results considering effects of the interventions on the microbiome measures were heterogeneous, both in reported outcomes and in the direction of effects.

### 4.1 Changes in Microbiome

As microbial diversity is thought to be lower among adults with obesity compared to lean adults [72], it could have been hypothesized that interventions aiming at improving weight or metabolic status would increase diversity. However, our systematic review found significant differences in both microbial *α*‐ and *β*‐diversity indices after intervention compared to placebo in only a small fraction of the studies, not systematically related to intervention type. Most of the studies reported no changes, implying that lifestyle and microbiota‐targeted interventions do not consistently affect gut microbiome diversity.

Interestingly, we found that dietary changes, especially with additional food supplementation, were associated with increases in both *α*‐ and *β*‐diversity of the gut microbiome. This finding aligns with a review by Puljiz et al. [12] who concluded that the dietary interventions exert impacts on gut microbiome quickly as gut microbiome is highly responsive to any changes within the gut. Supporting this, a landmark study by David et al. [73] showed that *β*‐diversity of participants changed within 24 h of adopting an animal‐based diet, but the microbial structure reverted to its original state within 48 h after the intervention ended. This rapid response highlighted the sensitivity of the gut microbiome to dietary changes, suggesting that microbial diversity might be particularly informative in measuring the impact of dietary intervention in obesity management. Our systematic review suggests that microbial diversity metrics could serve as early indicators of how dietary changes influence the gut microbiome, offering insights into personalized approaches for obesity treatment.

While microbial diversity is commonly regarded as an indicator of gut health in obesity, some studies in our review were associated with reduced diversity. This counterintuitive finding reflects how different interventions target the microbiome through distinct mechanisms. For instance, interventions with prebiotics and probiotics often aim to selectively promote the growth of certain beneficial taxa, which may lead to reduced overall diversity. In such cases, reduced diversity may indicate a targeted and beneficial compositional shift. This also highlights the limitations of relying on diversity indices alone to assess microbiome responses. Therefore, future studies to combine taxonomic profiling with functional metagenomics and host phenotyping are essential to obtain a more comprehensive understanding of how different interventions modulate the gut microbiome.

Notably, around half of the included studies did not report results on those indices, rendering it difficult to draw a comprehensive conclusion about the consistency of the effects of lifestyle and microbiota‐targeted interventions on the gut microbiome diversity. For instance, some prebiotic and probiotic studies did not report changes in microbial diversity presumably because it was hypothesized that those supplementary formulas selectively stimulate the growth of specific beneficial microbial taxa and may not induce overall changes in the microbial diversity. However, the lack of systematic reporting highlights a critical gap in the available microbiome‐RCT literature, as standardized assessment and consistent reporting of such widely used measures as microbial diversity indices [74] are crucial for cross‐study comparisons. The findings are helpful in underscoring the need for improved methodological rigor and uniformity in future studies to better understand the complex relationship between lifestyle and microbiota‐targeted interventions and the gut microbiome. Without such standardization, the field risks misestimating the potential impact of lifestyle and microbiota‐targeted interventions, thereby limiting their translation into effective public health strategies.

Other than the microbial diversity indices, we observed significant changes in abundances of specific microbial taxa after interventions. Among all intervention categories, prebiotic interventions were most frequently reported to cause changes in microbial abundance at the genus level in people with obesity. Particularly, the abundances of phylum Actinobacteria and genus *Bifidobacterium* were generally increased after prebiotic interventions, likely as a direct consequence of prebiotic consumption [75]. Prebiotic dietary fibers are complex, indigestible carbohydrates that are fermented into SCFAs by gut microbiota, including Actinobacteria and *Bifidobacterium* [13]. The increase in the aforementioned microbiota could lead to a higher production of SCFAs like butyrate and propionate, which in turn could stimulate the release of hormones such as satiety‐promoting peptide YY (PYY) and glucagon‐like peptide‐1 (GLP‐1), leading on the long run to reduced adiposity and overall weight gain [19]. This could also explain why most included prebiotic studies reporting diversity measures did not show changes therein. Prebiotic consumption might primarily modulate specific microbial taxa and functionality without significantly altering overall *α*‐ and *β*‐diversity metrics. Future studies should therefore complement diversity metrics with functional assessments such as metabolomics or transcriptomics to better capture intervention effects. Systematic exploration of prebiotic dosage effects could also reveal thresholds required to influence microbial diversity.

Results of four included probiotic interventional studies reported that an intervention‐induced growth of beneficial *Bifidobacterium* along with *Lactobacillus* was positively correlated with the reduction of body weight and fat [33, 34, 35, 76]. *Bifidobacterium* and *Lactobacillus* are common genera that are discussed to maintain gut health in obesity. A previous systematic review showed that the consumption of probiotics or synbiotics containing strains belonging to *Lactobacillus* and *Bifidobacterium* was associated with significant weight reductions in participants living with overweight and obesity [77]. Another RCT also showed that supplementation of the aforementioned genera was significantly associated with improved weight loss among 220 adults with obesity and hypercholesterolemia (−2.5%) [78].

The ratio of F/B has long been regarded as a hallmark of obesity, as a higher F/B ratio has been associated with increased energy harvest from food and enhanced fat storage, both key contributors to obesity. This suggests that an elevated F/B ratio may play a role in the development of obesity by promoting greater energy extraction and fat accumulation [79]. Supporting this, another systematic review of 32 studies demonstrated that individuals with obesity generally exhibit a higher F/B ratio compared to lean individuals [80]. This relationship has positioned the F/B ratio as a potential indicator of gut microbial imbalances linked to metabolic dysfunction. Our systematic review revealed that lifestyle and microbiota‐targeted interventions, particularly dietary interventions, led to either reduced or unchanged F/B ratios in people with obesity. These findings hint at the potentially positive impact of such interventions on gut microbial composition.

Nevertheless, only a small number of studies in our review reported changes in the F/B ratio, raising questions about its reliability and consistency as a biomarker. The limited reporting on F/B ratio changes may be attributed to the ongoing controversy regarding the use of F/B ratio as a marker of obesity. Evidence suggests that the F/B ratio is not universally consistent across different populations, dietary habits, and methodological approaches [79]. Factors such as variability in study designs, differences in sequencing technologies, and individual variations in response to dietary interventions further complicate its interpretation. These findings underscore the need for caution when interpreting the F/B ratio as a biomarker and highlight the importance of adopting a more comprehensive approach to studying gut microbial composition in obesity‐related research.

### 4.2 Changes in Microbial Metabolites

Other than microbial composition, changes in microbial metabolites like SCFAs have also been implicated in obesity and weight management [81]. A few studies in our systematic review reported increased SCFA levels, particularly with plant‐based diets like Mediterranean diet and supplementations of avocado, prebiotics, grains, and dairy products [38, 43, 47, 61, 82]. A similar trend was reported by a systematic review of 139 human and animal studies [83]. The systematic review demonstrated that high‐fat diet and Western diet were correlated with reduced SCFA levels while the supplementations of dietary fiber and probiotics were associated with increased SCFA concentrations. The authors suggested that diet could manipulate SCFA profile directly by supplying substrates for SCFA‐producing microbiota and indirectly by affecting microbiome composition [83].

However, most of the reported studies in our systematic review showed no change in both fecal and blood SCFAs after dietary interventions. The lack of effect could be attributed to methodological limitations, as SCFA levels can be influenced by sample collection methods, storage conditions, and analytical techniques, which can lead to variability across studies [84]. The variations in intervention designs, such as differences in diet composition, could contribute to inconsistent findings. Moreover, the duration of the studies might have been insufficient to observe detectable changes. Furthermore, differences in baseline microbiota composition, dietary adherence, and individual metabolic responses may contribute to inconsistent findings [85]. These factors highlight the complexity of interpreting SCFA levels in clinical trials and underscore the need for more standardized methodologies and longer‐duration studies. By addressing these limitations, it could provide a clearer picture of the role of SCFAs in obesity and their responsiveness to dietary interventions.

There was no consistent association between the levels of SCFAs and obesity measures observed in our review. This is somewhat surprising as another meta‐analysis of seven studies observed higher fecal concentrations of acetate, propionate, and butyrate among individuals with obesity compared to controls without obesity [86], which indicated a positive correlation between fecal SCFA levels and obesity. One plausible explanation for the inconsistency lies in the complex interplay between SCFA production, absorption, and utilization. Elevated fecal SCFA levels in individuals with obesity could reflect impaired SCFA absorption or altered gut barrier function, leading to reduced systemic availability despite increased production [87]. Alternatively, a higher abundance of SCFA‐producing bacteria in obesity might contribute to these elevated levels. This was supported by a study showed that the increased abundance of butyrate‐producing *Faecalibacterium* could raise the level of butyrate and increase the levels of appetite‐controlling glucagon‐like peptide 1 (GLP‐1) and peptide tyrosine (PPY) [88]. This may subsequently increase satiety, reduce food intake, and eventually contribute to improving obesity.

Cross‐sectional data from a RCT of our group also showed that fecal and serum SCFAs were inversely correlated with body fat mass in this subsample of young, overweight adults [89], though a 2‐week high‐dose prebiotics intake did not change those levels or replicate these correlations [21]. The failure to replicate the inverse correlation in the intervention study might indicate that short‐term interventions are insufficient to induce measurable changes in SCFA levels or their downstream effects on body composition. Alternatively, it could reflect individual variability in metabolic responses or the need for more targeted or prolonged interventions to elicit meaningful changes. The SCFA levels are influenced by various factors, including individual microbiome composition, dietary adherence, metabolic variability, and even methodological inconsistencies in measuring SCFAs [90]. Therefore, focusing solely on SCFA levels may oversimplify the intricate mechanisms underlying obesity and its management.

Taken together, while it can be hypothesized that SCFAs as microbial metabolites are implicated in obesity and subject to change upon microbiome‐changing interventions such as diets, most clinical trials in our systematic review do not report on, or do not support, a substantial effect of lifestyle and microbiota‐targeted interventions on increasing fecal or serum SCFA levels. Future studies should explore longer intervention durations, consider individual variability, and integrate microbiome data with other metabolic and inflammatory markers to better understand the mechanisms linking SCFAs to obesity and weight regulation.

Beyond SCFAs, only a few studies reported other microbial metabolites, such as bile acids and trimethylamine‐N‐oxide (TMAO), which were reported inconsistently and therefore were not systematically analyzed in this review. However, the microbial metabolites are increasingly recognized for their roles in host metabolism and obesity [91, 92]. Future reviews may consider systematically evaluating these additional metabolites to better understand microbiome‐mediated metabolic pathways.

### 4.3 Association Between Lifestyle and Microbiota‐Targeted Interventions and Obesity Measures

Based on our findings, among all kinds of interventions, dietary interventions were found to be relatively consistently linked to reduced body weight, body fat, and body circumference. Notably, not all the studies were aimed at weight loss on the short run; rather, they focused on inducing microbial changes. Thus, the findings of the systematic review reflect the impact on gut microbiome beyond the impact in body weight. Of note, consensus exists that dietary and exercise interventions are not sufficiently effective to induce clinically relevant weight loss in severe obesity in the long term, promoting adjunct alternative treatments such as incretins or bariatric surgery [93].

In our review, low‐calorie, low‐carbohydrate, low‐fat, and plant‐based diets were associated with significant weight reduction compared to exercise and FMT [48, 59, 65, 66, 67, 94]. This aligned with evidence synthesized by Medawar et al. [95], which highlighted the metabolic benefits of plant‐based diets, and Zhang et al. [96], which found low‐carbohydrate diets effective for weight loss. Additionally, reduced fat intake was identified as a safe and effective strategy for weight management [97].

Prebiotic studies in our review showed reductions in body fat, consistent with findings linking higher dietary fiber intake to improved weight status [97]. However, the shorter intervention periods (4 weeks–3 months) in our review may explain the less pronounced effects compared to studies with longer durations. Probiotic studies showed mixed results, likely due to short intervention periods (3–24 weeks). A meta‐analysis by Saadati et al. [98] demonstrated significant effects of probiotics on body weight, BMI, and body fat percentage only after longer interventions (15–40 weeks). Future research should explore optimal durations, formulas, and dosages for probiotics and prebiotics in obesity management.

Our systematic review observed that high‐intensity exercise increased body weight and reduced body fat after the interventional period, while there was also an increase in fat‐free mass. This was supported by a meta‐analysis of 16 RCTs that showed decreases in weight, BMI, and visceral fat after exercise interventions [99]. This aligns with WHO guidelines recommending 150–300 min of moderate aerobic activity weekly for health maintenance.

It is also suggested to have combination therapies for obesity, such as combining dietary and lifestyle interventions. For instance, a probiotic‐supplemented caloric reduced diet with exercise, showed significant reductions in weight, BMI, and body fat while increasing muscle mass [100]. Along with this, precision nutrition may further enhance obesity management. A phenotype‐tailored lifestyle intervention based on physiological and psychological assessments resulted in greater weight loss compared to standard approaches [101]. More RCTs are needed to validate the feasibility and effectiveness of personalized strategies.

### 4.4 Limitations

The present systematic review included a total of 87 randomized clinical trials. While additional database searches were deemed unlikely to identify further eligible studies, the literature search was limited to one database only (PubMed). While we aimed to include all relevant evidence through a comprehensive and systematic search strategy across PubMed, it is possible that there might be additional studies that were not identified. The predominance of dietary interventions among the included studies reflects the scope of the evidence available, rather than an intentional focus on diet. This may have indirectly resulted in a relative underrepresentation of other types of interventions, such as exercise and FMT. However, our methodology was designed to minimize selection bias and encompass a wide variety of intervention types to provide a balanced overview.

Additionally, some studies did not employ double‐blinding in their trials, which increased the risk of biases (i.e., attrition bias and reporting bias). Admittedly, double‐blinding could be challenging in lifestyle RCTs, for instance, it would be difficult to blind both participants and researchers in dietary interventions with different meals as well as exercises with different intensity. To overcome these limitations, some of the included studies involved independent personnel in the experiments, minimizing observer bias. Studies using supplements allocated the products by identical, opaque sachets for both intervention and placebo groups for concealment, offering active control conditions that can easily be blinded to both the participants and scientists.

Besides, not all included studies aimed for or controlled weight change as part of the intervention. Some studies targeted microbial changes specifically whereas some studies focused on changes in metabolic outcome more broadly. However, many studies did not report data on dietary counseling, meal plans, and physical activity monitoring, which made it challenging to determine whether the observed microbial changes resulted from the intervention or unintended energy balance alterations. Future studies should consider standardizing the reporting of dietary intake and energy balance‐related variables to better interpret the relationship between the interventions, caloric intake, and microbiome.

Moreover, the variability in the duration of the interventions was also a challenge in determining the optimal period for intervention. Other sources of heterogeneity, such as the variation of dietary components and control conditions, also led to discrepancies and limited the interpretation. For instance, control groups across the included studies ranged from habitual or unrestricted diets to active comparators like calorie‐restricted or macronutrient‐adjusted diets. These active‐control diets may have independently influenced microbial outcomes, potentially masking the true interventional effects and complicating the cross‐study comparisons. To improve interpretability and consistency across future trials, it is recommended to implement standardized control conditions where possible and clearly justify the expected microbial outcomes of the comparator diet.

The heterogeneity of reported outcomes across studies also limited the generalizability and comparability of the findings. Variations in outcome measures, intervention protocols, and study designs made it difficult to draw consistent conclusions about the effects of lifestyle interventions on the gut microbiome. For instance, not all studies assessed or reported associations between microbial changes and anthropometric outcomes such as body weight. The inconsistency in reported outcomes across the included studies made it more challenging to associate microbial changes with the effects of the interventions. Additionally, the use of fecal microbiome measures as the primary source of microbial data may not fully represent the entire gut microbiome. It is suggested that including microbial data from other sources, such as the oral microbiome, could provide a more comprehensive understanding of the role of human microbiome in obesity and metabolism [102].

Furthermore, the sample size in the included studies were different, ranging from 10 to 400 participants. This may affect the representation of the present study to reflect the significance of each interventional category. For instance, small sample sizes may reduce the statistical power and generalizability of findings, increase the risk of bias, and limit the ability to detect true effects of the interventions. This could potentially introduce positivity bias, where smaller studies are more likely to report significant results. Finally, the variation of sequencing methods across the studies, such as amplicon and shotgun metagenomic sequencing, which utilize different sequencing depth that amplified certain regions of interest, may have led to publication bias on microbial results. While we did not observe specific effects of sequencing method on the reported results, this was not systematically assessed in our review due to inconsistent reporting across studies. Thus, there might be potential methodological effects that could not be neglected. Future studies should further explore the impact of sequencing approaches on gut microbiome outcomes to enhance cross‐study comparability.

Despite the limitations, our systematic review could help to pave the way for a more tailored and effective approach to obesity treatment harnessing the potential of microbiome‐changing interventions. Our findings could lay a foundation for further precision nutrition approaches that can enhance the effectiveness of the existing treatment strategies for obesity. Most included studies focus on group‐level effects, which have limited our understanding of individual variability in response to lifestyle interventions. Future studies can be designed in a way where participants are stratified based on relevant modifiers like microbial composition and metabolic status. Various study designs like multi‐arm trials and crossover designs can be used to investigate how different interventions work for various subgroups within a population with overweight and obesity. Longitudinal studies can also be conducted to observe changes over time and understand how short‐ and long‐term dietary modifications affect individuals with obesity differently. Addressing these steps helps establish a solid foundation of robust data and refine dietary recommendations through real‐world settings.

## 5 Conclusion

In this systematic review of 87 RCTs ranging 2 weeks–1.5 years, lifestyle and microbiota‐targeted interventions across most of the studies, regardless of the intervention category, did not lead to significant changes in microbial *α*‐ or *β*‐diversity measures. However, most prebiotic interventions increased the abundance of Actinobacteria and *Bifidobacterium*, regarded as beneficial microbiota, which could promote the SCFA levels and maintain gut health. Probiotic interventions were also often effective in increasing the growth of *Lactobacillus* in the gut and reducing body weight and body fat.

However, results were not inconclusive and a meta‐analysis on methodologically harmonized outcomes may help to provide clearer insights. Gut microbiome studies often vary in methodologies, so it is challenging to draw firm conclusions due to the inconsistencies. By establishing standardized study protocols for assessing and reporting microbial outcomes, greater consistency and comparability of results could be obtained [103]. This would enhance the reliability of pooled data and facilitate robust meta‐analyses that reflect the true impact of gut microbiome on obesity.

Additionally, more mechanistic studies are required to uncover the underlying pathways through which specific diets and microbiome compositions interact with metabolic and immune pathways. This can help identify causal relationships and reveal specific microbial species or metabolites that are protective against obesity and related metabolic disorders. Ultimately, a shift from broad group‐level findings to more precise, personalized interventions in upcoming years is thought to improve nutritional science by customizing dietary plans based on individual's unique microbial profile, ultimately supporting more effective and sustainable health outcomes.

## Author Contributions

Y.T.L., A.A., E.M., and A.V.W. contributed to the conceptualization of the study and developed the study protocol. Y.T.L., A.A., and D.B.Ö. contributed to data extraction. Y.T.L. and A.A. provided the manuscript draft. Y.T.L., D.B.Ö., E.M., D.E.A.J., A.V., and A.V.W. contributed to writing‐review and editing. All authors read and approved the final manuscript.

## Funding

This work was funded by German Research Foundation (DFG) (209933838, WI 3342/3–1), and by the Max Planck Society.

## Conflicts of Interest

The authors declare no conflicts of interest.

## Supporting information


**Supplementary File 1:** Full PubMed search strategy, including search terms, filters, date ranges, and MeSH term corrections used for study selection.
**Table S1:** PRISMA Checklist: A detailed checklist following the PRISMA guidelines for systematic reviews, outlining the key reporting items and their corresponding sections in the manuscript.
**Table S2a:** Study characteristics for studies with ≥ 3 interventions.
**Table S2b:** Study characteristics of a study with five intervention groups.
**Table S2c:** Study characteristics of a study with five intervention groups and a control group.
**Table S3:** Changes in obesity‐related measures and microbiome outcomes in each intervention study.
**Table S4:** Assessment criteria for risk of bias (ROB).

## Data Availability

Data sharing is not applicable to this article as no datasets were generated or analyzed during the current study. This work is a systematic review of existing research and does not report new raw data. The study protocol was preregistered in PROSPERO (CRD42021281444) and is available at https://www.crd.york.ac.uk/PROSPERO/view/CRD42021281444.
