# Supplementary material for: Impacts of Lifestyle and Microbiota‐Targeted Interventions for Overweight and Obesity on the Human Gut Microbiome: A Systematic Review
Source: Obes Rev. 2025 Dec 10;27(3):e70037. doi: 10.1111/obr.70037 (PMC12926627; doi:10.1111/obr.70037)
Supplement: Supplementary file 1 — Full PubMed search strategy, including search terms, filters, date ranges, and MeSH term corrections used for study selection. Table S1: PRISMA Checklist: A detailed checklist following the PRISMA guidelines for systematic reviews, outlining the key reporting items and their corresponding sections in the manuscript. Table S2a: Study characteristics for studies with ≥ 3 interventions. Table S2b: Study characteristics of a study with five intervention groups. Table S2c: Study characteristics of a study with five intervention groups and a control group. Table S3: Changes in obesity‐related measures and microbiome outcomes in each intervention study. Table S4: Assessment criteria for risk of bias (ROB). [file OBR-27-e70037-s001.pdf]

Impacts of lifestyle and microbiota-targeted interventions for overweight and obesity on the human gut microbiome: a systematic review

Yee Teng Lee[1,2*], Ayça Akan[2,3], Dilara Beyza Önel[2], Evelyn Medawar[2], Daria E. A. Jensen[1,2], Arno Villringer[1,2], A. Veronica Witte[1,2*]

[1]Clinic for Cognitive Neurology, University of Leipzig Medical Center, Leipzig, Germany
[2]Department of Neurology, Max Planck Institute of Human Cognitive and Brain Sciences, Leipzig, Germany
[3]Berlin School of Mind and Brain, Humboldt University of Berlin, Berlin, Germany

*Corresponding authors

Correspondence:

***Yee Teng Lee**, Department of Neurology, Max Planck Institute of Human Cognitive and Brain Sciences, Stephanstraße 1A, 04103 Leipzig, Germany, leey@cbs.mpg.de

*A. Veronica Witte, Cognitive Neurology, University of Leipzig Medical Center & Department of Neurology, Max Planck Institute of Human Cognitive and Brain Sciences, Stephanstraße 1A, 04103 Leipzig, Germany, witte@cbs.mpg.de

Supplementary File 1: Full PubMed search strategy, including search terms, filters, date ranges, and MeSH term corrections used for study selection.

Full PubMed search query:

Search:((adiposity) OR (obesity) OR (overweight)) AND ((gut microbiota) OR (gut microbiome)) AND ((diet therapy) OR (weight loss) OR (fecal microbiota transplantation) OR
(exercise) OR (intervention) OR (probiotics) OR (prebiotics)) Filters: Clinical Trial, Randomized Controlled Trial, Humans, English, Aged: 65+ years, Adult: 19+ years, Young
Adult: 19-24 years, Adult: 19-44 years, Middle Aged + Aged: 45+ years, Middle Aged: 45-64
years, 80 and over: 80+ years, from 23/10/2011 – 23/10/2021

Additional search with same inclusion and exclusion criteria: from 24/10/2021 – 31/05/2024

MeSH term corrections (performed in the first search):

Gastrointestinal Microbiome (MeSH) and Gut microbiota + Gut microbiome (free term) comparison:

Gut Microbiome OR Gut Microbiota: 204 articles

Gastrointestinal Microbiome: 140 articles

Gastrointestinal Microbiome NOT IN Gut Microbiome OR Gut Microbiota: 0 articles

Gut Microbiome OR Gut Microbiota covers all the articles under Gastrointestinal Microbiome.

Therefore, **Gut Microbiome OR Gut Microbiota free terms** rather than the MeSH term Gastrointestinal Microbiome was assumed.

Table S1 PRISMA Checklist: A detailed checklist following the PRISMA guidelines for systematic reviews, outlining the key reporting items and their corresponding sections in the manuscript

Section and Topic	Item #	Checklist item	Location where item is reported
**TITLE**			
Title	1	Identify the report as a systematic review.	Page 1
**ABSTRACT**			
Abstract	2	See the PRISMA 2020 for Abstracts checklist.	Page 2
**INTRODUCTION**			
Rationale	3	Describe the rationale for the review in the context of existing knowledge.	Page 2-4
Objectives	4	Provide an explicit statement of the objective(s) or question(s) the review addresses.	Page 4
**METHODS**			
Eligibility criteria	5	Specify the inclusion and exclusion criteria for the review and how studies were grouped for the syntheses.	Page 5
Information sources	6	Specify all databases, registers, websites, organisations, reference lists and other sources searched or consulted to identify studies. Specify the date when each source was last searched or consulted.	Page 4-5
Search strategy	7	Present the full search strategies for all databases, registers and websites, including any filters and limits used.	Page 4-5, Table 1
Selection process	8	Specify the methods used to decide whether a study met the inclusion criteria of the review, including how many reviewers screened each record and each report retrieved, whether they worked independently, and if applicable, details of automation tools used in the process.	Page 4-5
Data collection process	9	Specify the methods used to collect data from reports, including how many reviewers collected data from each report, whether they worked independently, any processes for obtaining or confirming data from study investigators, and if applicable, details of automation tools used in the process.	Page 5-6
Data items	10a	List and define all outcomes for which data were sought. Specify whether all results that were compatible with each outcome domain in each study were sought (e.g. for all measures, time points, analyses), and if not, the methods used to decide which results to collect.	Page 6
	10b	List and define all other variables for which data were sought (e.g. participant and intervention characteristics, funding sources). Describe any assumptions made about any missing or unclear information.	Page 6
Study risk of bias assessment	11	Specify the methods used to assess risk of bias in the included studies, including details of the tool(s) used, how many reviewers assessed each study and whether they worked independently, and if applicable, details of automation tools used in the process.	Page 5-6
Effect measures	12	Specify for each outcome the effect measure(s) (e.g. risk ratio, mean difference) used in the synthesis or presentation of results.	N/A
Synthesis methods	13a	Describe the processes used to decide which studies were eligible for each synthesis (e.g. tabulating the study intervention characteristics and comparing against the planned groups for each synthesis (item #5)).	Page 5-6
	13b	Describe any methods required to prepare the data for presentation or synthesis, such as handling of missing summary statistics, or data conversions.	N/A
	13c	Describe any methods used to tabulate or visually display results of individual studies and syntheses.	Page 5-6
	13d	Describe any methods used to synthesize results and provide a rationale for the choice(s). If meta-analysis was performed, describe the model(s), method(s) to identify the presence and extent of statistical heterogeneity, and software package(s) used.	Page 5-6

Section and Topic	Item #	Checklist item	Location where item is reported
	13e	Describe any methods used to explore possible causes of heterogeneity among study results (e.g. subgroup analysis, meta-regression).	N/A
	13f	Describe any sensitivity analyses conducted to assess robustness of the synthesized results.	N/A
Reporting bias assessment	14	Describe any methods used to assess risk of bias due to missing results in a synthesis (arising from reporting biases).	Page 5-6
Certainty assessment	15	Describe any methods used to assess certainty (or confidence) in the body of evidence for an outcome.	N/A
**RESULTS**			
Study selection	16a	Describe the results of the search and selection process, from the number of records identified in the search to the number of studies included in the review, ideally using a flow diagram.	Page 6-7, Figure 1
	16b	Cite studies that might appear to meet the inclusion criteria, but which were excluded, and explain why they were excluded.	N/A
Study characteristics	17	Cite each included study and present its characteristics.	Page 7-8, Table S2a-c
Risk of bias in studies	18	Present assessments of risk of bias for each included study.	Page 12, Table S4
Results of individual studies	19	For all outcomes, present, for each study: (a) summary statistics for each group (where appropriate) and (b) an effect estimate and its precision (e.g. confidence/credible interval), ideally using structured tables or plots.	Figure 3, Table S3
Results of syntheses	20a	For each synthesis, briefly summarise the characteristics and risk of bias among contributing studies.	Page 6-12
	20b	Present results of all statistical syntheses conducted. If meta-analysis was done, present for each the summary estimate and its precision (e.g. confidence/credible interval) and measures of statistical heterogeneity. If comparing groups, describe the direction of the effect.	Figure 3
	20c	Present results of all investigations of possible causes of heterogeneity among study results.	N/A
	20d	Present results of all sensitivity analyses conducted to assess the robustness of the synthesized results.	N/A
Reporting biases	21	Present assessments of risk of bias due to missing results (arising from reporting biases) for each synthesis assessed.	N/A
Certainty of evidence	22	Present assessments of certainty (or confidence) in the body of evidence for each outcome assessed.	N/A
**DISCUSSION**			
Discussion	23a	Provide a general interpretation of the results in the context of other evidence.	Page 12-18
	23b	Discuss any limitations of the evidence included in the review.	Page 18-20
	23c	Discuss any limitations of the review processes used.	Page 18-20
	23d	Discuss implications of the results for practice, policy, and future research.	Page 20
**OTHER INFORMATION**			
Registration and protocol	24a	Provide registration information for the review, including register name and registration number, or state that the review was not registered.	Page 5
	24b	Indicate where the review protocol can be accessed, or state that a protocol was not prepared.	Page 5
	24c	Describe and explain any amendments to information provided at registration or in the protocol.	N/A
Support	25	Describe sources of financial or non-financial support for the review, and the role of the funders or sponsors in the review.	Page 1, 21

Section and Topic	Item #	Checklist item	Location where item is reported
Competing interests	26	Declare any competing interests of review authors.	Page 1, 21
Availability of data, code and other materials	27	Report which of the following are publicly available and where they can be found: template data collection forms; data extracted from included studies; data used for all analyses; analytic code; any other materials used in the review.	N/A

Note: N/A, not applicable.

Table S2a Study characteristics for studies with ≥3 interventions

Category	Study	Population (Overweight / Obese)	Age range (years)	Total number of study subjects	Type of intervention				Duration of intervention	Sequencing method
					Intervention 1	Intervention 2	Intervention 3	Control /Placebo		
**Probiotics**	Antonopoulou et al., 2024	Overweight	48.6 ± 9.3	92	150 g enriched yogurt fortified with an olive oil pomace extract (OOPLE)	one serving of plain yogurt every day (150 g)	N/A	at most one yogurt every 14 days	8 weeks	real-time PCR
	Brahe et al., 2015	Obese	40-70	58	Mixture of *L. Paracasei* F19 ($9.4x10^{10}$ CFU/dose) and maltodextrin and placebo buns	Flaxseed mucilage buns and maltodextrin	N/A	Placebo buns and maltodextrin	6 weeks	shotgun metagenomics
	Crovesy et al., 2021[@]	Obese	25-45	51	Probiotic (capsules containing 10^9 CFU of *B. lactis* UBBLa-70 and a sachet with 5 g of maltodextrin)	Synbiotic (capsules containing 10^9 CFU of *B. lactis* UBBLa-70 and a sachet with 5 g of fructooligosaccharides)	N/A	Capsules with gelatin and a sachet of 5 g with maltodextrin	8 weeks	M/D
	Danshiitsoodol et al., 2022	Overweight	55.5 ± 11.1	80	Capsules containing mixtures of dextrin, 1.3×10^5 cells of heat-killed IJH-SONE68 strain-containing powder (260 mg) and cultured broth	N/A	N/A	Placebo capsules of dextrin	12 weeks	16S rRNA gene sequencing

Gomes et al., 2020	Both	20-64	60	Probiotic mixture containing 2 × 10^{10} CFU/day of *L. acidophilus* LA-14, *L. casei* LC-11, *Lactococcus lactis* LL-23, *B. bifidum* BB-06, and *B. lactis* BL-4, and maltodextrin	N/A	N/A	200 mg maltodextrin	8 weeks	16S rDNA gene sequencing
Hibberd et al., 2019	Both	48.2 (10.4)	134	Probiotic *B. animalis subsp. lactis* 420™ (B420), 10^{10} CFU/day in 12 g microcrystalline cellulose	Synbiotic LU and B420, 10^{10} CFU/day of B420 in 12 g/day LU	Prebiotic Litesse® Ultra™ polydextrose (LU), 12 g/day	12 g/day of microcrystalline cellulose	24 weeks	16S rRNA gene sequencing
Janczy et al., 2020	Both	40.8(14)	59	Sanprobi Super Formula® (*B. lactis* W51-52, *L. acidophilus* W22, *L. paracasei* W20, *L. plantarum* W21, *L. Salivarius*, *Lactococus lactis*, Inulin, Fructooligosacchari des)	N/A	N/A	Placebo capsules	12 weeks	M/D
Pedret et al., 2019	Obese	44.9	135	*B. animalis subsp. lactis* CECT 8145 (Ba8145) (100 mg of the live strain, 10^{10} CFU/capsule containing 200 mg of maltodextrin)	Heat killed Ba8145 (100 mg of heat-killed CECT8145 strain (10^{10} CFU before the heat treatment/capsule containing 200 mg of maltodextrin)	N/A	300 mg of maltodextrin	12 weeks	16S rRNA gene sequencing

	Rahayu et al., 2021	Overweight	35-56	60	Milk powder with *L. plantarum* Dad-13 (2 × 10^9 CFU)	N/A	N/A	Milk powder from a local supermarket	90 days	16S rRNA gene sequencing
	Sergeev et al., 2020	Both	47.4	20	Probiotics (*L. acidophilus* DDS-1, *B. lactis* UABla-12, *B. longum* UABl-14, and *B. bifidum* UABb-10) and prebiotics (5.5 g/day of trans-galactooligosaccharide (GOS) mixture)	N/A	N/A	Low-carbohydrate and high-protein diet with reduced energy intake	12 weeks	16S rRNA gene sequencing
	Simon et al., 2015	Lean vs. Obese	40-65	10	Capsules containing *L. reuteri* SD5865 (2 X 10^{10} viable cells)	N/A	N/A	Placebo capsules	8 weeks	16S rRNA gene sequencing
	Sohn et al., 2022	Overweight	46.7 ± 10.9	81	2×10^9 CFU of L. plantarum K50 capsules (total 4 × 10^9 CFU/day)	N/A	N/A	Unspecified placebo	12 weeks	shotgun metagenomics
**Prebiotics**	Canfora et al., 2017	Both	58.8 (7.2)	46	Galacto-oligosaccharride (GOS) powder that contained GOS (69% GOS, 23% lactose, 5% glucose and galactose, and 3% moisture)	N/A	N/A	Isocaloric maltodextrin	12 weeks	16S rRNA microarray using HITChip (for the Human Intestinal Tract Chip)
	Chambers et al., 2019	Both	60(1)	12	Inulin-propionate ester	inulin	N/A	Cellulose	42 days	16S rRNA gene sequencing

Christensen et al., 2020*	Overweight	18-60*	29	Arabinoxylan oligosaccharides (AXOS)	N/A	N/A	Polyunsaturated fatty acids	4 weeks	16S rRNA gene sequencing + shotgun metagenomics
Dewulf et al., 2013	Obese	18-65*	44	Mixture of inulin and oligofructose	N/A	N/A	Maltodextrin	3 months	real-time PCR + 16S rRNA microarray
Hess et al., 2020	Both	48.6 (8.4)	116	Fiber supplement	N/A	N/A	Maltodextrin	12 weeks	16S rRNA gene sequencing
Kjølbæk et al., 2020	Both	18-60*	29	Arabinoxylan oligosaccharides (AXOS)	N/A	N/A	Polyunsaturated fatty acids (fish oil capsules (about 228 kJ/d) containing 3.6 g/d n-3 PUFA (1.32 g/d DHA and 1.86 g/dEPA)	12 weeks	16S rRNA gene sequencing
Lambert et al., 2017	Both	20-62	50	Wafers containing 5 g/serving of yellow pea fiber	N/A	N/A	Isocaloric wafers without pea fiber	12 weeks	16S rRNA gene sequencing
Medawar et al., 2024	Overweight	28.3 ± 6.55	59	30 g inulin per day	N/A	N/A	16 g maltodextrin	2 weeks	16S rRNA gene sequencing

	Rebello et al., 2015	Both	48(2) /45-50	30	Gastrointestinal Microbiome Modulator (GIMM) containing 4 g inulin, 2.5 g oat β-glucan, blueberry extract without sugar	N/A	N/A	placebo (0 g β-glucan, 8.8 g total fiber)	4 weeks	M/D
	Reimer et al., 2017	Both	37.8 (1.6)	125	Prebiotic bar containing inulin-type fructans (6 g oligofructose and 2 g chicory root)	Protein bar containing 5 g whey protein	Combination bar containing 8 g inulin-type fructans and 5 g whey protein	Isocaloric control bar (unspecified)	12 weeks	16S rRNA gene sequencing
	Salden et al., 2018	Both	48(16)	80	7.5 g Arabinoxylans powder	15 g Arabinoxylans powder	N/A	15 g Maltodextrin	6 weeks	16S rRNA gene sequencing + 16S rDNA gene sequencing
	Vulevic et al., 2013	Overweight	18-65*	48	Galactooligosaccharide mixture (B-GOS)	N/A	N/A	Maltodextrin	12 weeks	16S rRNA-targeted fluorescence in situ hybridization (FISH) analysis
**Grain supplementation**	Christensen et al., 2019*	Overweight	30-65*	46	Whole grain wheat	whole grain rye	N/A	Refined wheat	6 weeks	16S rRNA gene sequencing
	Dotimas et al., 2024	Overweight	26.5 ± 6.9	40	Wheat germ supplements (30 g wheat germ, 10 g peanut butter, 10 g	N/A	N/A	Control meal (30 g corn meal, 10 g peanut butter, 10 g honey,	4 weeks	16S rRNA gene sequencing

					honey, and 5 g powdered milk)			and 5 g powdered milk)		
	Kopf et al., 2018	Both	32.3 (12.4)	52	Whole grains	Fruit and vegetables	N/A	Refined grain products	6 weeks	16S rRNA gene sequencing
	Roager et al., 2019	Overweight	20-65*	60	Whole grain diet (>75 g/day)	N/A	N/A	Refined grain diet (<10 g/day)	8 weeks	16S rRNA gene sequencing + shotgun metagenomics
	Schutte et al., 2018	Overweight	61	50	98 g whole grain wheat	N/A	N/A	98 g refined wheat	12 weeks	16S rRNA gene sequencing
	Sheflin et al., 2017	Both	37-84	37	30 g of heat-stabilized rice bran	35 g of cooked navy bean powder	N/A	Macronutrient matched control	4 weeks	16S rRNA pyrosequencing
	van Trijp et al., 2021	Both	45-70	50	Whole Grain Wheat	N/A	N/A	Colored refined wheat	12 weeks	16S rRNA gene sequencing
	Vuholm et al., 2017	Overweight	41.6-60.4	75	Whole grain rye (WGR)	Whole grain wheat (WGW)	N/A	Refined wheat (RW)	6 weeks	16S rRNA gene sequencing
**Dairy product supplementation**	Fernandez-Raudales et al., 2012	Both	32(7)	81	Low glycinin soymilk	Conventional soymilk	N/A	Bovine milk	3 months	16S rRNA gene sequencing

	Vors et al., 2020	Both	56-62	58	5 g Milk Polar Lipid	3 g Milk Polar Lipid	N/A	full fat cream cheese without polar lipid	4 weeks	16S rDNA gene sequencing
**Protein**	Beaumont et al., 2017	Overweight	18-45	42	Plant protein	Animal protein	N/A	Maltodextrin	3 weeks	16S rDNA gene sequencing
	Bel Lassen et al., 2021	Both	18-65*	107	High protein product	N/A	N/A	Normoproteic product	12 weeks	16S rRNA gene sequencing + shotgun metagenomics
	Sun et al., 2022	Both	61.3 ± 7.7	60	Whey protein sachet (7.6 g protein/10 g)	N/A	N/A	Whey protein hydrolysate sachet (8.4 g hydrolysate protein/10 g)	8 weeks	16S rRNA gene sequencing
**Mixed diet**	Basciani et al., 2020	Obese	50-70	48	Whey protein-based very-low-calorie ketogenic diet	Vegetable protein-based very-low-calorie ketogenic diet	N/A	Animal protein-based very-low-calorie ketogenic diet	45 days	16S rRNA gene sequencing
	Dao et al., 2016	Both	41.9 (12.3)	49	Calorie-restricted diet enriched with fibers and protein	N/A	N/A	Individually customized weight-maintenance diet	6 weeks	16S rRNA gene sequencing
	Diao et al., 2023	Obese	42.5 ± 9.8	120	Low-calorie DASH diet	Low calorie diet	N/A	Non-calorie restricted diet	12 weeks	real-time PCR

Dong et al., 2020	Both	55.8 (10.7)	80	Calorie-restricted high protein diet (HPD) (30% protein, 40% carbohydrate, 30% fat by calorie intake)	N/A	N/A	Calorie-restricted normal protein diet (NPD) (15% protein, 55% carbohydrate, 30% fat)	8 weeks	16S rRNA gene sequencing
Grembi et al., 2020	Obese	41.5 (6.3)	122	Low carbohydrate diet	N/A	N/A	Low fat diet	10 weeks	16S rRNA gene sequencing
Haro et al., 2016	Obese	63.3 (2.0)	20	Mediterranean diet	N/A	N/A	Low-fat, high-complex carbohydrate diet	1 year	16S rRNA gene sequencing
Hjorth et al., 2020[#]	Both	44.2 (13.3)	62	New Nordic diet (high in dietary fiber, whole grain, fruit, and vegetables)	N/A	N/A	Average Danish Diet (average Western diet)	26 weeks	16S rRNA gene sequencing + real-time PCR
Johnstone et al., 2020	Both	20-62	19	Weight-loss diet consisting of 30% protein, 30% fat, and 40% carbohydrates	Weight-maintenance diet with resistant starch	N/A	Control weight-maintenance diet with digestible starch	49 days	16S rRNA gene sequencing + real-time PCR
Kahleova et al., 2020	Both	55.2 (11.2)	168	Low-fat vegan diet (vegetables, grains, legumes, fruit) restricted to 20-30 g daily fat intake, supplemented with 500 µg/day of vitamin B12	N/A	N/A	Normal diet	16 weeks	16S rRNA gene sequencing

Ma et al., 2021	Both	47.9 ± 6.6	48	Low-carbohydrate diet (20 g/day in the first week and slowly added up weekly until 120 g/day)	N/A	N/A	Traditional Chinese diet with a pre-trial daily energy intake reduced to up 35%	12 weeks	shotgun metagenomics
Marungruang et al., 2018	Obese	18-60*	47	Multifunctional diet (soybean and soy protein-based products, barley, rye, blue berries, cinnamon, plant stanol-containing margarine, fish, vinegar, rapeseed oil, almond, whey protein, guar gum)	N/A	N/A	Dietary fiber-providing food like processed cereals, white wheat bread, dark wheat bread, fruits and vegetables	8 weeks	16S rRNA gene sequencing
Meslier et al., 2020	Both	42.5 (12.5)	82	Mediterranean Diet	N/A	N/A	Regular diet	8 weeks	16S rRNA gene sequencing
Mohr et al., 2024	Both	50.2 ± 2.3	41	Supplements and snacks with total calorie intake restricted to 1350-1500 kcals/day and 1700-1850 kcals/day for women and men respectively	N/A	N/A	Mediterranean diet consisting of fresh vegetables, fruits, nuts and legumes with total caloric intake restricted to 1200 and 1500 kcals/day for women and men respectively	8 weeks	16S rRNA gene sequencing + shotgun metagenomics

	Sowah et al., 2022	Both	50.2 ± 8.0	147	Intermittent calorie restriction	Continuous calorie restriction	N/A	Healthy balanced diet	50 weeks	16S rRNA gene sequencing
	Swarte et al., 2020	Overweight	58.9 (4.3)	46	High-dairy diet	N/A	N/A	Low-dairy diet	6 weeks	16S rRNA gene sequencing
	Vitale et al., 2021	Both	20-60	30	Mediterranean Diet	N/A	N/A	Western-type diet	8 weeks	16S rRNA gene sequencing
	Zhang et al., 2021	Both	47.8 ± 10.0	51	Low-carbohydrate diet (10% to 25% of total energy intake)	N/A	N/A	Normal diet without energy restriction group	12 weeks	16S rDNA gene sequencing
**Additional food supplementation**	Bendtsen et al., 2018	Obese	44(1)	80	High calcium intake	N/A	N/A	Low calcium intake	24 weeks	16S rRNA gene sequencing
	Bratlie et al., 2021	Both	18-69	76	Cod fish	Salmon	N/A	No fish intake	8 weeks	16S rRNA gene sequencing
	Choo et al., 2021	Both	60.8 (7.4)	69	Raw almond snacks	N/A	N/A	Isocaloric, high carbohydrate biscuit snacks	8 weeks	16S rRNA gene sequencing
	de Souza et al., 2015	Both	23-59 / 38.5	33	L-glutamine	N/A	N/A	L-alanine	2 weeks	16S rRNA gene sequencing
	Fava et al., 2022	Both	47.2 ± 9.9	67	Aleurone-supplemented meal: One portion of bread (2 × 58 g buns containing 18	N/A	N/A	Cellulose-supplemented meal: one portion of bread (2 × 58 g	4 weeks	16S rRNA gene sequencing

				g aleurone/portion), one portion of biscuits (2 × 15 g biscuits containing 9 g aleurone/portion) and one portion of breakfast cereals (36 g of ready to eat cereals, containing 9 g aleurone/portion)			buns containing 18 g cellulose/portion), one portion of biscuits (2 × 15 g biscuits containing 9 g cellulose/portion) and one portion of breakfast cereals (36 g of ready to eat cereals, containing 9 g cellulose/portion)		
Gao et al., 2021	Overweight	21.4 (2.8)	117	Fried meat	N/A	N/A	Boiled, steamed meat or served with sauce dressing	4 weeks	16S rRNA gene sequencing
González-Sarrías et al., 2017	Both	40-65	60	Pomegranate extract capsule (450 mg containing 160 mg phenolics)	Pomegranate extract (1.8 g containing 640 mg phenolics)	N/A	Maltodextrin	6 weeks	M/D
Han et al., 2015	Obese	30-60	24	Fresh kimchi	N/A	N/A	Fermented kimchi	8 weeks	16S rRNA gene sequencing
Hiel et al., 2020	Obese	51 (10.4)	150	Inulin coupled with fructan-enriched vegetables	N/A	N/A	Maltodextrin coupled with low fructan vegetables	3 months	16S rRNA gene sequencing

Hochkogler et al., 2017	Overweight	20-41	19	Sachet (containing milk powder, refined sugar, lamequick® CE 1 SF, aroma, and xanthan gum) added with 0.05 mg nonivamide	N/A	N/A	Sachet with same product formulation as intervention, but without nonivamide	12 weeks	16S rRNA gene sequencing
Li et al., 2022	Both	30.9 ± 6.4	69	Konjaku flour	N/A	N/A	Lotus root starch	5 weeks	Shotgun metagenomics
Moreno-Indias et al., 2016	Lean vs. Obese	44(15) / 18-70	20	272 mL red wine	N/A	N/A	272 mL de-alcoholized red wine	60 days	16S rRNA gene sequencing
Most et al., 2017	Overweight	55	37	Capsule containing epigallocatechin-3-gallate and resveratrol (EGCG+RES)	N/A	N/A	Microcrystalline cellulose capsule	12 weeks	M/D
Naderpoor et al., 2019	Both	51-72	32	Vitamin D	N/A	N/A	Unspecified placebo	16 weeks	16S rRNA gene sequencing
Payahoo et al., 2019	Obese	20-65 / 41(12)	60	2 capsules containing 125 mg of oleoylethanolamide (OEA)	N/A	N/A	Capsules containing 125 mg of starch	8 weeks	16S rRNA gene sequencing
Roach et al., 2022¥	Overweight	54 ± 10	64	4 g dose of seaweed extract, Ulva sp. 84-derived sulfated polysaccharide “xylorhamnoglucuronan” (SXRG84)	2 g dose of SXRG84	N/A	Unspecified placebo	6 weeks	16S rRNA gene sequencing
		52 ± 13	64	2 g dose of SXRG84	N/A	N/A	Unspecified placebo	12 weeks	

	Santamarina et al., 2024	Both	56.5 ± 5.5	41	Silymarin-enriched supplements containing prebiotics, minerals and herbal medicine Silybum marianum	N/A	N/A	Supplements containing prebiotics and minerals without silymarin	180 days	16S rRNA gene sequencing
	Song et al., 2015	Obese	36 (6.8)	40	Drink containing Schisandra chinensis fruit	N/A	N/A	Drink without Schisandra chinensis fruit	12 weeks	real-time PCR + 16S rRNA gene sequencing
	Thompson et al., 2021	Overweight	35 (0.5)	157	Isocaloric meals with avocado	N/A	N/A	Isocaloric meal	12 weeks	16S rRNA gene sequencing
**Mixed-type intervention**	Gutiérrez-Repiso et al., 2019	Obese	18-65*	33	Synbiotic group: Very-low-calorie ketogenic diet (VLCKD) with synbiotics (capsules containing *B. lactis*, *L. rhamnosus*, *B. longum* ES1 and prebiotic fiber) in Phase 1; Low-calorie diet (LC) with capsules containing *B. animalis* subsp. *lactis* and prebiotic fiber in Phase 2	Placebo-synbiotic group: VLCKD with placebo in Phase 1; LC diet with synbiotics in Phase 2 (capsules containing *B. animalis* subsp. *lactis* and prebiotic fiber)	N/A	Placebo (in both Phases 1 and 2)	4 months	16S rRNA gene sequencing

Hric et al., 2021	Both	48.1 (12.9)	22	Reduced caloric diet supplemented with probiotic Bryndza cheese and moderate-to-vigorous aerobic exercise	N/A	N/A	Reduced caloric diet and moderate-to-vigorous aerobic exercise	4 weeks	16S rDNA gene sequencing
Lee et al., 2014	Both	19-65*	50	3 g of Bofutsushosan herbal extract and probiotic capsules (5 billion viable cells of *Streptococcus thermophiles* (KCTC 11870BP), *L. plantarum* (KCTC 10782BP), *L. acidophilus* (KCTC 11906BP), *L. rhamnosus* (KCTC 12202BP), *B. lactis* (KCTC 11904BP), *B. longum* (KCTC 12200BP), and *B. breve* (KCTC 12201BP))	N/A	N/A	3 g of Bofutsushosan herbal extract and placebo capsules without bacterial strains	8 weeks	real-time PCR
Rajkumar et al., 2014	Overweight	40-60	60	Probiotic capsules containing bifidobacteria (*B. longum*, *B. infantis*, and *B. breve*), lactobacilli (*L. acidophilus*, *L. paracasei*, *L. delbrueckii* subsp. *bulgaricus*, and *L. plantarum*), and *S. salivarius* subsp. *thermophilus*	Omega-3 capsules (180 mg EPA and 120 mg DHA per capsule)	Both probiotic and omega-3 capsules	40 mg of microcrystalline cellulose	6 weeks	M/D

	Meir et al., 2021	Obese	51.1 ± 10.5	294	Calorie-restricted Mediterranean diet rich in vegetables, poultry and fish and 28 g/day of walnuts as well as physical activity	Green Mediterranean diet enriched with plants and polyphenols, 3-4 cups/day of green tea, 100 g/day of frozen Wolffia globosa plant frozen cubes as green shake, as well as physical activity	N/A	Followed healthy dietary guidelines combined with physical activity	18 months	16S rRNA gene sequencing
	Muralidharan et al., 2021	Both	55-75	400	Energy-restricted Mediterranean diet, physical activity promotion, and behavioural support (IG)	N/A	N/A	Unrestricted-caloric Mediterranean diet	1 year	16S rRNA gene sequencing
	Rinott et al., 2021	Obese	52.4 (10.8)	90	Phase 1: Mediterranean diet Phase 2: FMT capsules	Phase 1: Green Mediterranean diet Phase 2: FMT capsules	N/A	Phase 1: Following healthy dietary guidelines Phase 2: Capsules containing agarose in normal saline/glycerol	14 months	shotgun metagenomics
**Exercise**	Cullen et al., 2024	Both	28.5 ± 4.2	32	Supervised resistance training of 18 sessions (3 days/week)	N/A	N/A	Based on the provided handout of the recommended age-fit levels of physical activity	6 weeks	16S rRNA gene sequencing
	Dupuit et al., 2022	Both	60.9 ± 4.8	17	Supervised high-intensity interval training and resistance training	N/A	N/A	No training	12 weeks	16S rRNA gene sequencing

					(HIIT + RT) program three times per week (approximately 45 minutes each)				
	Kern et al., 2020	Both	20-45	130	Bike commuting	Moderate exercise	Vigorous exercise	Habitual Living	24 weeks	16S rRNA gene sequencing
**Fecal microbiota transplantation (FMT)**	Yu et al., 2020	Obese	25-60	24	Oral FMT capsules from healthy donors	N/A	N/A	Powdered cocoa and gelatin mixed with a solution of 40% (v/v) glycerol and normal saline	12 weeks	16S rDNA gene sequencing
	Zhang et al., 2024	Obese	47.8 ± 10.0	29	FMT + fermentable fiber	FMT + cellulose	Placebo FMT + fermentable fiber	Placebo FMT + cellulose	6 weeks	16S rRNA gene sequencing

@, the study reported correlations between microbiota and metabolites; ¥, the article published separate findings of two studies; *, studies stratified study subjects according to *P*/*B* ratio; #, the study stratified study subjects according to salivary amylase gene copy number and reported specific results related to *Prevotella*, *Bacteroides* and *P*/*B* ratio only. N/A, not applicable; M/D, missing data.

Note for Age range: Mean (standard deviation); *,eligibility range if age range was not provided.

Table S2b Study characteristics of a study with five intervention groups

Category	Study	Population (Overweight /Obese)	Age range (years)	Total number of study subjects	Type of intervention					Duration of intervention	Sequencing method
					Intervention 1	Intervention 2	Intervention 3	Intervention 4	Intervention 5		
**Mixed Diet**	Fava et al., 2013	Both	55.6 (9.9)	130	High monosaturated fat diet with high glycemic index (HM/HGI)	High monosaturated fat diet with low glycemic index (HM/LGI)	High carbohydrate diet with high glycemic index (HC/HGI)	High carbohydrate diet with low glycemic index (HC/LGI)	High saturated fat diet (HS)	24 weeks	16S rRNA gene sequencing

See legend under Table S2a.

Table S2c Study characteristics of a study with five intervention groups and a control group

Category	Study	Population (Overweight /Obese)	Age range (years)	Total number of study subjects	Type of intervention						Duration of intervention	Sequencing method
					Intervention 1	Intervention 2	Intervention 3	Intervention 4	Intervention 5	Control / Placebo		
**Probiotics**	Krumbeck et al., 2018	Obese	44.3 (11.2)	114	*B. adolescentis* IVS-1 (IVS-1; Probiotics)	*B. animalis* subsp. *lactis* BB-12 (BB-12; Probiotics)	IVS-1 + galactooligosaccharides (prebiotics; GOS)	BB-12 + GOS	GOS	Lactose	3 weeks	16S rRNA gene sequencing

See legend under Table S2a.

Table S3 Changes in obesity-related measures and microbiome outcomes in each intervention study

Category	Study	Change in body weight	Change in body fat	Change in other anthropometric measures	Change in microbial diversity		Change in microbial abundance																	Change in levels of SCFAs
							Phylum				Family		Genus											
					α-diversity	β-diversity	Actinobacteria	Bacteroidetes	Firmicutes	Proteobacteria	Lachnospiraceae	Ruminococcaceae	*Akkermansia*	*Bacteroides*	*Bifidobacterium*	*Clostridium*	*Faecalibacterium*	*Lactobacillus*	*Prevotella*	*Roseburia*	*Ruminococcus*	*Streptococcus*	
**Probiotics**	Antonopoulou et al., 2024																						
	Brahe et al., 2015																						
	Crovesy et al., 2021@																						
	Danshiitsoodol et al., 2022																						
	Gomes et al., 2020																						
	Hibberd et al., 2019																						
	Janczy et al., 2020																						
	Krumbeck et al., 2018																						
	Pedret et al., 2019																						
	Rahayu et al., 2021																						
	Sergeev et al., 2020																						
	Simon et al., 2015																						
	Sohn et al., 2022																						
**Prebiotics**	Canfora et al., 2017																						
	Chambers et al., 2019																						
	Christensen et al., 2020*																						
	Dewulf et al., 2013																						
	Hess et al., 2020																						
	Kjølbæk et al., 2020																						
	Lambert et al., 2017																						

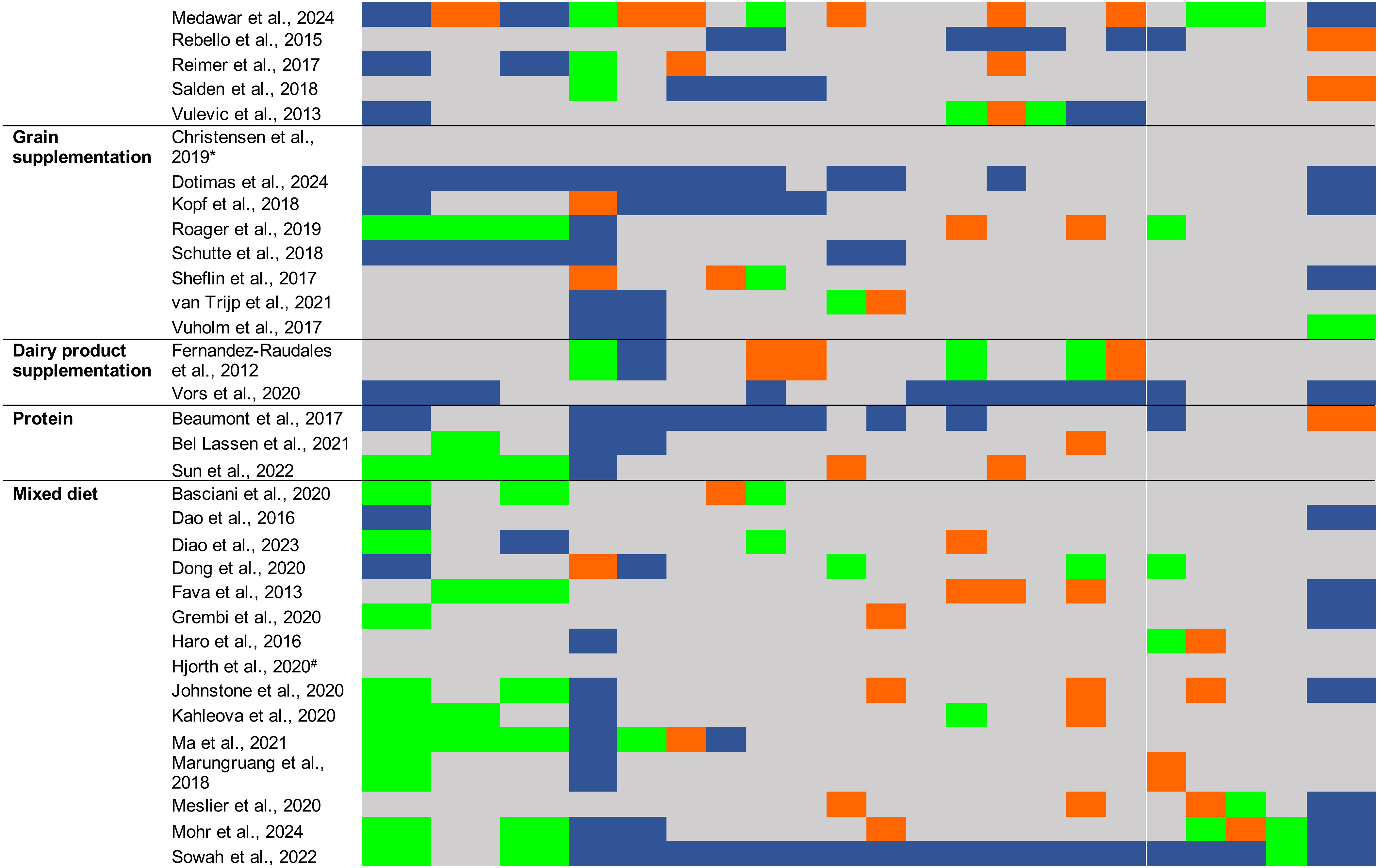
Medawar et al., 2024
Rebello et al., 2015
Reimer et al., 2017
Salden et al., 2018
Vulevic et al., 2013
Grain supplementation
Christensen et al., 2019*
Dotimas et al., 2024
Kopf et al., 2018
Roager et al., 2019
Schutte et al., 2018
Sheflin et al., 2017
van Trijp et al., 2021
Vuholm et al., 2017
Dairy product supplementation
Fernandez-Raudales et al., 2012
Vors et al., 2020
Protein
Beaumont et al., 2017
Bel Lassen et al., 2021
Sun et al., 2022
Mixed diet
Basciani et al., 2020
Dao et al., 2016
Diao et al., 2023
Dong et al., 2020
Fava et al., 2013
Grembi et al., 2020
Haro et al., 2016
Hjorth et al., 2020#
Johnstone et al., 2020
Kahleova et al., 2020
Ma et al., 2021
Marungruang et al., 2018
Meslier et al., 2020
Mohr et al., 2024
Sowah et al., 2022

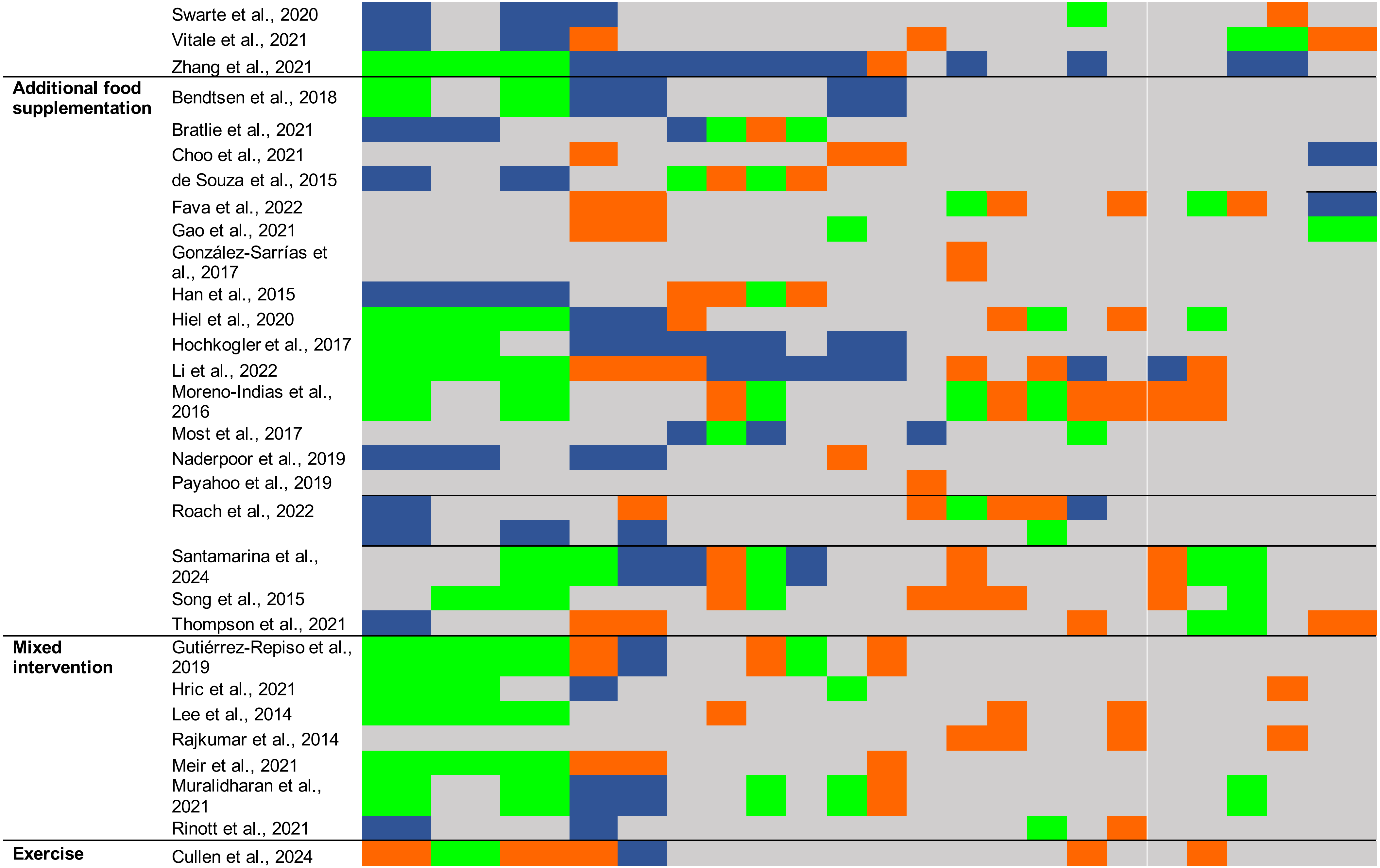
Swarte et al., 2020
Vitale et al., 2021
Zhang et al., 2021
Additional food supplementation
Bendtsen et al., 2018
Bratlie et al., 2021
Choo et al., 2021
de Souza et al., 2015
Fava et al., 2022
Gao et al., 2021
González-Sarrías et al., 2017
Han et al., 2015
Hiel et al., 2020
Hochkogler et al., 2017
Li et al., 2022
Moreno-Indias et al., 2016
Most et al., 2017
Naderpoor et al., 2019
Payahoo et al., 2019
Roach et al., 2022
Santamarina et al., 2024
Song et al., 2015
Thompson et al., 2021
Mixed intervention
Gutiérrez-Repiso et al., 2019
Hric et al., 2021
Lee et al., 2014
Rajkumar et al., 2014
Meir et al., 2021
Muralidharan et al., 2021
Rinott et al., 2021
Exercise
Cullen et al., 2024

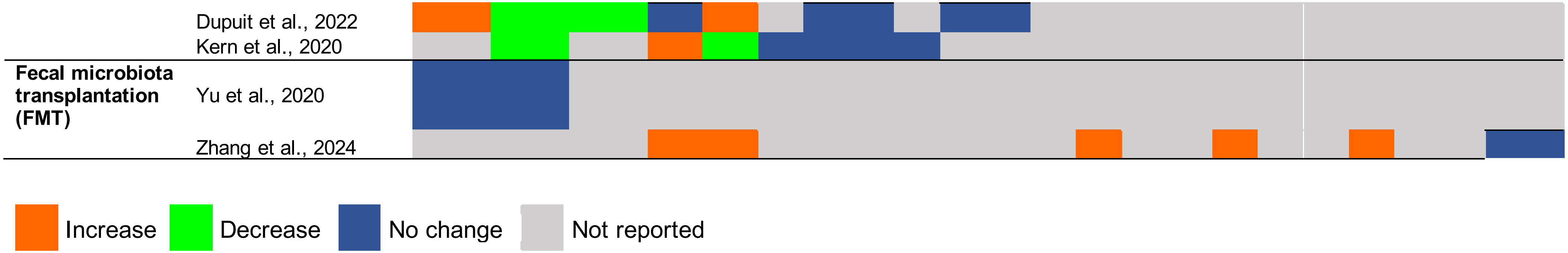

Note: @, the study reported correlations between microbiota and metabolite; *, the studies stratified study subjects according to *P*/*B* ratio; #, the study stratified study subjects according to salivary amylase gene copy number and reported specific results related to *Prevotella*, *Bacteroides* and *P*/*B* ratio only; ϕ, the study contained findings of two separate experiments that were conducted on the same group of participants.

Table S4 Assessment criteria for risk of bias (ROB)

Category	Study	Selection bias			Attrition Bias		Conflict of Interest	Pre-registration	ROB Score
		Random sequence generation	Allocation Concealment	Blinding of Participants and Personnels	Other Bias	Incomplete Outcome data			
Probiotics	Antonopoulou et al., 2024	-	-	D	-	-	-	-	very low
	Brahe et al., 2015	-	-	S	-	-	-	-	very low
	Crovesy et al., 2021	-	-	D	-	+	-	-	very low
	Gomes et al., 2020	-	-	D	-	+	-	-	very low
	Hibberd et al., 2019	?	?	D	-	-	+	-	low
	Janczy et al., 2020	?	?	D	+	-	-	+	moderate
	Krumbeck et al., 2018	?	?	D	-	-	+	-	low
	Pedret et al., 2019	-	-	D	+	+	+	-	moderate
	Rahayu et al., 2021	?	-	D	-	-	-	-	very low
	Sergeev et al., 2020	?	-	S	+	-	-	-	low

	Simon et al., 2015	?	-	D	+	-	+	-	moderate
	Sohn et al., 2022	?	?	?	?	-	-	-	low
Prebiotics	Canfora et al., 2017	-	-	D	-	-	-	-	very low
	Chambers et al., 2019	-	-	D	-	-	-	-	very low
	Christensen et al., 2020	?	?	?	?	-	-	-	low
	Dewulf et al., 2013	?	-	D	-	-	-	-	low
	Hess et al., 2020	-	-	D	-	+	-	-	very low
	Kjølbæk et al., 2020	-	+	+	?	-	-	-	moderate
	Lambert et al., 2017	-	-	D	-	-	-	-	very low
	Medawar et al., 2024	-	-	D	-	-	-	-	very low
	Rebello et al., 2015	-	-	D	-	-	-	-	very low
	Reimer et al., 2017	-	-	D	-	+	-	-	low
	Salden et al., 2018	-	-	D	-	-	-	-	very low
	Vulevic et al., 2013	+	?	D	?	-	-	-	low

Grain	Christensen et al., 2019	-	-	S	-	-	-	-	very low
	Dotimas et al., 2024	-	-	S	-	-	-	-	very low
	Kopf et al., 2018	-	?	?	-	-	-	-	very low
	Roager et al., 2019	-	-	D	-	-	-	-	very low
	Schutte et al., 2018	-	-	D	-	-	-	-	very low
	Sheflin et al., 2017	+	?	S	-	+	-	-	moderate
	van Trijp et al., 2021	-	?	D	-	+	-	-	low
	Vuholm et al., 2017	-	+	S	-	?	-	-	very low
Dairy product supplementatio n	Fernandez-Raudales et al., 2012	-	?	D	-	?	+	+	moderate
	Vors et al., 2020	-	?	D	-	-	-	-	very low
Protein	Beaumont et al., 2017	-	-	D	-	-	-	-	very low
	Bel Lassen et al., 2021	-	?	D	+	?	-	-	low
	Sun et al., 2022	-	-	S	-	-	-	-	very low
Mixed diet	Basciani et al., 2020	-	?	+	+	-	-	-	moderate

Dao et al., 2016	+	+	+	-	-	-	-	moderate
Diao et al., 2023	-	-	S	-	-	-	-	very low
Dong et al., 2020	-	-	D	-	+	-	-	very low
Fava et al., 2013	-	?	S	-	+	-	-	very low
Grembi et al., 2020	?	?	?	-	+	-	-	moderate
Haro et al., 2016	?	?	?	+	-	-	-	moderate
Hjorth et al., 2020	?	?	?	-	-	-	-	low
Johnstone et al., 2020	?	?	?	?	-	?	-	moderate
Kahleova et al., 2020	-	?	S	-	-	-	-	very low
Ma et al., 2021	-	?	S	-	-	-	-	very low
Marungruang et al., 2018	-	?	?	-	-	-	-	very low
Meslier et al., 2020	-	-	D	-	-	-	-	very low
Mohr et al., 2024	+	+	?	?	-	-	-	moderate
Sowah et al., 2022	-	?	S	?	?	-	-	low

	Swarte et al., 2020	-	?	+	?	-	-	-	low
	Vitale et al., 2021	-	-	S	-	-	-	-	very low
	Zhang et al., 2021	-	-	S	?	-	-	-	very low
Additional food supplementation	Bendtsen et al., 2018	-	?	?	-	+	-	-	low
	Bratlie et al., 2021	-	-	S	-	-	-	-	very low
	Choo et al., 2021	-	-	S	-	-	-	-	very low
	de Souza et al., 2015	?	-	D	-	+	-	+	moderate
	Fava et al., 2022	-	-	D	-	-	-	-	very low
	Gao et al., 2021	-	-	S	-	-	-	-	very low
	González-Sarrías et al., 2017	-	-	D	-	-	-	-	very low
	Han et al., 2015	?	?	?	-	-	-	+	moderate
	Hiel et al., 2020	-	-	S	-	+	-	-	very low
	Hochkogler et al., 2017	?	?	S	-	-	+	+	moderate

	Li et al., 2022	-	?	D	-	-	-	+	low
	Moreno-Indias et al., 2016	?	?	?	-	-	-	+	moderate
	Most et al., 2017	?	-	D	-	-	-	-	very low
	Naderpoor et al., 2019	-	?	D	-	-	-	-	very low
	Payahoo et al., 2019	?	-	D	-	-	-	-	very low
	Roach et al., 2022	-	-	D	+	-	+	-	low
	Santamarina et al., 2024	-	?	D	?	-	-	-	very low
	Song et al., 2015	?	-	D	-	+	-	-	low
	Thompson et al., 2021	-	?	S	-	-	-	-	very low
Mixed intervention	Gutiérrez-Repiso et al., 2019	-	?	S	-	+	-	-	low
	Hric et al., 2021	?	?	?	-	?	-	+	
	Lee et al., 2014	?	?	D	-	-	-	-	very low
	Rajkumar et al., 2014	?	-	S	-	+	-	-	low
	Meir et al., 2021	-	+	?	?	-	+	-	moderate

	Muralidharan et al., 2021	-	-	D	-	-	-	-	very low
	Rinott et al., 2021	-	?	D	-	-	-	-	very low
Exercise	Cullen et al., 2024	-	+	?	?	-	-	-	low
	Dupuit et al., 2022	+	+	S	?	-	-	-	moderate
	Kern et al., 2020	-	-	?	-	+	-	-	low
Fecal microbiota transplantation (FMT)	Yu et al., 2020	-	-	D	-	-	+	-	very low
	Zhang et al., 2024	-	-	D	-	-	-	-	very low

-,low risk; +, high risk; ?, uncertain risk; S, single blind; D, double blind.
